# Zinc‐Mediated Lysosomal Destabilization Links Mitochondrial Damage to Neuronal Death in a Cellular MPP ^+^ Model of Parkinson's Disease

**DOI:** 10.1111/jnc.70363

**Published:** 2026-02-01

**Authors:** Hyun‐Seung Lee, Sun‐Ah Kang, Jae‐Won Eom, Min Seong Kim, Ji‐Soo Kim, Yang‐Hee Kim

**Affiliations:** ^1^ Department of Integrative Bioscience and Biotechnology Sejong University Seoul Republic of Korea; ^2^ Zincure Corp Seoul Republic of Korea; ^3^ Institute of Bioscience and Biotechnology Sejong University Seoul Republic of Korea

**Keywords:** lysosomal membrane permeabilization (LMP), mitochondria, Parkinson's disease, reactive oxygen species (ROS), zinc

## Abstract

Dysregulation of autophagy and lysosomal function is central to Parkinson's disease (PD), yet the upstream mechanisms leading to lysosomal failure remain unclear. Across primary mouse cortical neurons, MT‐3 deficient primary mouse astrocytes, human iPSC‐derived midbrain dopaminergic neurons, and Rho^0^ CHO cells lacking mitochondrial respiration, we investigated how mitochondrial stress perturbs zinc (Zn^2+^) homeostasis and lysosomal integrity. We identify intracellular zinc as a critical mediator linking mitochondrial dysfunction to lysosomal membrane permeabilization (LMP) and neuronal death. Inhibition of mitochondrial complex I by 1‐methyl‐4‐phenylpyridinium (MPP^+^) elevated reactive oxygen species (ROS) and intracellular zinc, jointly driving LMP. Blocking either ROS or zinc markedly attenuated lysosomal damage and cell death, demonstrating that both act upstream of LMP. To define zinc regulation, we examined metallothionein‐3 (MT‐3), a brain‐enriched zinc‐binding protein. MT‐3‐deficient astrocytes were more vulnerable to MPP^+^ and zinc overload (ZnCl_2_) but paradoxically resistant to hydrogen peroxide (H_2_O_2_), suggesting that MT‐3 buffers cytosolic zinc during mitochondrial injury or extracellular zinc influx yet can release bound zinc under oxidative conditions. Using Rho^0^ cells, we show that MPP^+^ toxicity depends on mitochondrial ROS, as loss of mitochondrial function nearly abolished cell death. However, Rho^0^ cells were highly sensitive to ZnCl_2_ and H_2_O_2_ and exhibited markedly reduced lysosomal abundance, indicating limited capacity to sequester zinc and increased susceptibility to zinc‐mediated injury. These findings support a coordinated system in which lysosomes and zinc‐binding proteins maintain zinc homeostasis. When cytosolic zinc rises, its accumulation within lysosomes induces LMP and accelerates cell death. Collectively, our results identify intracellular zinc as an upstream trigger of lysosomal dysfunction and neurodegeneration. Zinc‐mediated LMP provides a mechanistic link between mitochondrial injury, impaired autophagic flux, and α‐synuclein pathology in PD. Enhancing zinc homeostasis and lysosomal resilience may offer promising therapeutic strategies.

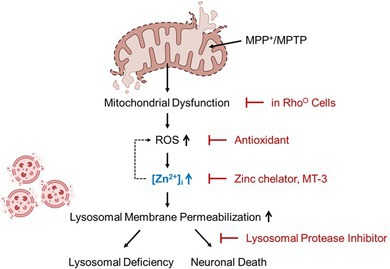

## Graphical abstract

When mitochondria are damaged by MPP^+^, they produce excess reactive oxygen species (ROS) and release zinc (Zn^2+^) inside the cell. This surge in zinc overwhelms lysosomes—the cell's major zinc‐buffering organelles—causing their membranes to break down. Leaking lysosomal enzymes then trigger neuronal death. Treatments that reduce ROS, bind zinc, or block lysosomal proteases strongly protect against this damage. Cells lacking mitochondrial respiration do not show ROS or zinc increases and are resistant to MPP^+^, highlighting mitochondria as the starting point of this process. Because repeated lysosomal injury can diminish lysosomal capacity, these events may also contribute to protein accumulation seen in neurodegeneration.

**Abbreviations**
6‐OHDA: 6‐hydroxydopamine
BAX: BCL2‐associated X protein
BSA: bovine serum albumin
CHO: Chinese Hamster Ovary
CTSB: cathepsin B
DCF: H2DCFDA
FZ: FluoZin‐3
GCase: glucocerebrosidase
hiPSCs: human induced pluripotent stem cells
iPSCs: induced pluripotent stem cells
KO: knock‐out
Leu: leupeptin
LMP: lysosomal membrane permeabilization
LT: LysoTracker Red
mDA: midbrain dopaminergic
MitoT: MitoTEMPO
MMP: mitochondrial membrane potential
MPP^+^
: 1‐methyl‐4‐phenylpyridinium
MS: MitoSOX
MT‐3: metallothionein‐3
PD: Parkinson's disease
PI: propidium iodide
ROS: reactive oxygen species
RRID: Research Resource Identifier
SNpc: substantia nigra pars compacta
TFEB: transcription factor EB
TH: tyrosine hydroxylase
WT: wild‐type

## 1 Introduction

Parkinson's disease (PD) is a prevalent neurodegenerative disorder that affects a significant portion of the elderly population. The hallmark of PD is the loss of dopaminergic neurons in the substantia nigra pars compacta (SNpc) region of the brain and the accumulation of Lewy bodies, intraneuronal proteinaceous cytoplasmic inclusions (Dauer and Przedborski 2003). The pathogenesis of PD is multifactorial and involves a complex interplay between genetic and environmental factors. Genetic mutations, such as those affecting the *SNCA* gene that encodes α‐synuclein, a major component of Lewy bodies (Stefanis 2012), as well as *Parkin*, *PINK1*, and *DJ‐1 genes* involved in the mitochondrial quality control system (Trempe and Fon 2013; Mata et al. 2004; Tan and Skipper 2007), and the *GBA* gene that encodes glucocerebrosidase (GCase) leading to lysosomal storage disease (Bae et al. 2015; Yang et al. 2013; Gegg and Schapira 2018), have been implicated in PD development. Moreover, exposure to pesticides and other environmental toxins is also known to contribute to mitochondrial dysfunction and oxidative stress, thereby increasing the risk of PD (Dick 2006; Subramaniam and Chesselet 2013). Therefore, a comprehensive understanding of the underlying mechanisms of PD pathogenesis, especially mitochondrial damage and lysosomal dysfunction, is necessary for developing effective therapeutic interventions (Guerra et al. 2019).

The involvement of oxidative stress in dopaminergic neuronal death in PD has also been suggested. The presence of catecholaminergic neurotoxin 6‐hydroxydopamine (6‐OHDA) in the midbrain of PD patients has been reported, and its injection has been utilized as an animal model of PD (Borah and Mohanakumar 2012; Glinka et al. 1997; Schober 2004). 6‐OHDA induces an increase of reactive oxygen species (ROS) as well as dopaminergic neuronal death (Borah and Mohanakumar 2012). Similarly, Complex I inhibitors, rotenone or 1‐methyl‐4‐phenylpyridinium (MPP^+^), increase ROS production and lead to the death of dopaminergic nerve cells (Borah and Mohanakumar 2012; Glinka et al. 1997; Schober 2004; Li et al. 2003; Shimoke et al. 2003; Giordano et al. 2012). The injection of these toxic substances into animals causes damage to dopaminergic neurons and the appearance of PD symptoms (Blesa and Przedborski 2014), indicating a close association between ROS and dopaminergic neuronal death in PD.

Zinc is an essential trace metal required for the maintenance of cellular and organismal viability. Proteomic analyses have shown that approximately 10% of all proteins bind zinc, underscoring its broad regulatory influence on protein structure and function (Andreini et al. 2006; Hara et al. 2017). Oxidative stress induces the release of zinc from proteins, resulting in elevated cytosolic zinc (Slepchenko et al. 2017; Lee et al. 2010). The typical concentration of free cytosolic zinc ranges from 100 to 250 pM, and an increase to approximately 10 nM is sufficient to induce cell death (Pratt et al. 2021; Maret 2017; Jiang et al. 2001; Tuncay and Turan 2020). Once cytosolic free zinc rises, cells regulate its levels not only through buffering by zinc‐binding proteins such as MT‐3 but also by sequestering zinc into intracellular organelles, including mitochondria and lysosomes, to maintain zinc homeostasis (Sensi, Ton‐That, Sullivan, et al. 2003; Kukic et al. 2014). However, excessive zinc accumulation imposes substantial stress on these organelles—particularly lysosomes, which sequester the elevated zinc—and can compromise their integrity. Excessive zinc loading disrupts lysosomal structure and triggers lysosomal membrane permeabilization (LMP), ultimately leading to cell death (Medvedeva et al. 2009; McCord and Aizenman 2014; Lee and Koh 2010; Liu et al. 2021). Consistent with this, several studies have reported that treatment with MPP^+^ or 6‐OHDA increases ROS and intracellular zinc levels (Lee et al. 2009; Rojas et al. 2005; Sheline et al. 2010, 2013; Hwang et al. 2008). H_2_O_2_ likewise elevates cytoplasmic zinc, which rapidly translocates into lysosomes and induces LMP and cell death (Hwang et al. 2008). Lysosomal disruption due to LMP, followed by dopaminergic neuronal loss, has been observed in an MPTP‐induced animal model of PD (Dehay et al. 2010). However, the mechanistic link between mitochondrial damage and lysosomal vulnerability remains unclear.

This study aimed to investigate whether MPP^+^ induces LMP in neuronal cultures and whether zinc plays a vital role in this process. To propose a zinc‐mediated mechanism of lysosomal dysfunction and neuronal damage, we examined the role of zinc in MPP^+^‐induced neuronal death and explored the involvement of metallothionein‐3 (MT‐3) and intracellular organelles in regulating labile zinc homeostasis. Our findings suggest that zinc acts as a critical link between mitochondrial damage and lysosomal destabilization, ultimately contributing to MPP^+^‐induced neurotoxicity.

## 2 Materials and Methods

### 2.1 Mouse Cerebrocortical Cultures

As previously described, cerebrocortical cultures, including neurons and astrocytes, were prepared from embryonic day 13–14 mice (Kim et al. 1999). Pregnant ICR female mice (Orient Bio, Gyeonggi‐do, South Korea; RRID:IMSR_CRL:022) were euthanized by rapid cervical dislocation without anesthesia (Total number of mice ≈120), in accordance with protocols approved by the Institutional Animal Care and Use Committee (IACUC) of Sejong University (SJ‐20230119). Embryos were immediately euthanized by decapitation without anesthesia. All procedures were performed by trained personnel certified in each method. This approach was chosen to eliminate potential confounding effects of anesthetic agents on embryonic brain tissues. Embryonic brains were promptly dissected, and for each litter, all embryo brains from the same dam were pooled before dissociation and plating. Dissociated cortical cells were seeded onto poly‐D‐lysine (Sigma‐Aldrich, Merck KGaA, Darmstadt, Germany; Cat. No. P0899)‐coated plates (SPL Life Sciences, Gyeonggi‐do, South Korea), 3.5‐4 hemispheres per plate. Primary cerebrocortical cultures were maintained in a 5% growth medium [glutamine‐free Dulbecco's modified Eagle medium (DMEM, Gibco, Thermo Fisher Scientific, Waltham, MA, USA; Cat. No. 11960‐044; RRID:SCR_013551) with 25 mM glucose, 44 mM sodium bicarbonate, 2 mM glutamine, 5% fetal bovine serum (HyClone, Cytiva, Marlborough, MA, USA; Cat. No. SH30919.03), and 5% horse serum (Gibco; Cat. No. 16050‐122)] at 37°C in a humidified 5% CO_2_ atmosphere. All experiments were performed at DIV 10–14.

All animal experimental procedures were conducted in accordance with the Guidelines for the Care and Use of Laboratory Animals and were reviewed and approved by the IACUC of Sejong University (SJ‐20230119).

Cerebrocortical astrocyte cultures of wild type or Metallothionein‐3 knockout (MT‐3 KO) mice were kindly provided by Dr. Jae‐Young Koh (University of Ulsan College of Medicine, South Korea). According to the providing laboratory, 3‐day‐old neonatal mice (P3 pups) were euthanized by decapitation without anesthesia (Total number of mice ≈20), in accordance with protocols approved by the IACUC of Asan Institute for Life Sciences, University of Ulsan College of Medicine (Approval No.: 2019‐12‐343). For each preparation, cerebrocortical tissues from all pups within the same litter were pooled prior to dissociation to minimize inter‐individual variability. Dissociated cerebrocortical cells were cultured in a 14% growth medium [glutamine‐free DMEM (Gibco) with 25 mM glucose, 44 mM sodium bicarbonate, 2 mM glutamine, 7% fetal bovine serum (HyClone), and 7% horse serum (Gibco)] at 37°C in a humidified 5% CO_2_ atmosphere until confluence was reached. Upon reaching 100% confluence, the growth medium was replaced with DMEM containing 5% serum. The confluent astrocyte cultures were used for experiments in our laboratory within a few days of receipt.

### 2.2 Human Induced Pluripotent Stem Cell Cultures (hiPSCs)

Human induced pluripotent stem cells (hiPSCs) used in this study were the hFSiPS3‐1 line, which was officially obtained from the Korea National Stem Cell Bank (MCB; Korea National Institute of Health, Republic of Korea) under a material transfer agreement (contract No. 2024‐0046). The hFSiPS3‐1 line was originally generated from human dermal fibroblasts (ScienCell, #2320) by Sendai virus–mediated reprogramming using the CytoTune‐iPS Reprogramming Kit (Invitrogen; Cat. No. A16517; RRID:SCR_013100) with OCT3/4, SOX2, KLF4, and c‐MYC. STR profiling confirmed the cell‐line identity, and all microbial contamination assays, including mycoplasma PCR, were negative. G‐banded karyotyping (46,XY) and CNV‐chip analysis revealed no chromosomal abnormalities. Pluripotency was validated by alkaline phosphatase staining, immunocytochemistry of OCT4, TRA‐1‐60, TRA‐1‐81, and SSEA4, qRT‐PCR analysis of pluripotency markers, embryoid‐body differentiation, and teratoma formation. hiPSCs were maintained under feeder‐free conditions on vitronectin (VTN‐N; Thermo Fisher Scientific, Cat. No. A14700; RRID:SCR_013217)‐coated plates in Essential 8 (E8) medium (Thermo Fisher Scientific, Cat. No. A1517001; RRID:SCR_014829). Culture plates were prepared by coating with recombinant human vitronectin at 5 μg/mL in DPBS (WellGene, Korea; Cat. No. LB001‐02) and incubating for 1 h at 37°C.

### 2.3 Midbrain Dopaminergic Neuronal Differentiation

iPSCs were dissociated with Accutase (Sigma‐Aldrich, Cat. No. A6964; RRID:SCR_013283) and plated onto Geltrex (Gibco; Cat. No. A1413202)‐coated plates. Cells were maintained in a KSR‐based medium consisting of DMEM/F‐12 (Welgene; Cat. No. LM002‐04), 20% KnockOut Serum Replacement (Gibco; Cat. No. 10828028; RRID:SCR_013317), MEM‐NEAA (Welgene; Cat. No. LS005‐01), L‐glutamine (Welgene; Cat. No. LS002‐01), β‐mercaptoethanol (Sigma‐Aldrich, Cat. No. M7522), 10 ng/mL FGF2 (ACRO Biosystems, Beijing, China; Cat. No. 30214535), and 10 μM Y‐27632 (Cayman Chemical, Cat. No. 10005583; RRID:SCR_021083). When cultures reached ~90% confluency (Day 0), neural induction was initiated by replacing the medium with KSR containing 200 nM LDN193189 (Stemcell Technologies, Canada; Cat. No. 72149) and 10 μM SB431542 (BioGems, USA; Cat. No. 3014193). From Days 1–7, 100 ng/mL SHH (ACRO Biosystems, Cat. No. SH7‐H5229), 2 μM purmorphamine (MedChemExpress, Monmouth Junction, NJ, USA; Cat. No. HY‐15108), and 100 ng/mL FGF8 (ACRO Biosystems, Cat. No. FGB‐H5115) were added, and 3 μM CHIR99021 (Tocris Bioscience, Bristol, UK; Cat. No. 4423; RRID:SCR_002000) was included from Day 3 to Day 11 to promote ventral midbrain identity. Beginning on Day 5, cultures were gradually transitioned from KSR to N2 (Gibco; Cat. No. 17502048; RRID:SCR_013748) medium in 25% increments every other day, completing the shift by Day 11. On Day 11, neural progenitors were dissociated and replated onto Geltrex‐coated plates in an expansion medium containing DMEM/F‐12, N‐2 supplement, L‐glutamine, penicillin–streptomycin (Welgene; Cat. No. LS202‐02), LDN193189, CHIR99021, and Y‐27632. For midbrain dopaminergic neuron (mDA) maturation, cells were transferred to Neurobasal (Welgene; Cat. No. LM022‐01)/B‐27 (Gibco; Cat. No. 12587010; RRID:SCR_013778) medium supplemented with CHIR99021, BDNF (ACRO Biosystems, Cat. No. BDF‐H5219), Ascorbic acid (Sigma‐Aldrich, Cat. No. A4544), GDNF (ACRO Biosystems, Cat. No. GDF‐H5219), TGF‐β3 (Gibco; Cat. No. PHG9305), dbcAMP (MedChemExpress, Cat. No. HY‐B0764), and DAPT (BioGems International, USA; Cat. No. 2088055), and maintained for at least 10 additional days to generate midbrain dopaminergic neurons.

### 2.4 Generating Rho^0^ Cells

The parental Chinese Hamster Ovary (CHO) cell line (RRID:CVCL_0213) is not listed as a misidentified or cross‐contaminated cell line in the ICLAC database. Rho^0^ cells were generated beginning at passage 5 by culturing CHO cells in medium containing 1 μg/mL ethidium bromide (Promega Corporation, Madison, WI, USA; Cat. No. H5041), following established procedures for mtDNA depletion (King and Attardi 1989; Chomyn 1996). Because mitochondrial DNA–deficient cells require exogenous pyrimidines, Rho^0^ cells were maintained in Ham's F‐12 medium (WELGENE, Gyeongsangbuk‐do, South Korea; Cat. No. LM010‐02) supplemented with 10% FBS, 50 μg/mL uridine (MP Biomedicals LLC, Solon, OH, USA; Cat. No. 103216), 0.025 M glucose (Sigma‐Aldrich; Cat. No. G7021), and 1% gentamicin (Duchefa Biochemie, Haarlem, The Netherlands; Cat. No. G0124), consistent with previous reports describing uridine dependence in Rho^0^ cells (Hu et al. 2000). All experiments were performed between passages 20 and 30. No formal STR‐based authentication was performed, as validated STR panels for hamster‐derived cell lines are not widely available.

### 2.5 Treatment With MPP ^+^ and Other Chemicals/Drugs

Prior to treatment with drugs, the cultures were washed with serum‐free medium [Eagle's minimal essential medium (MEM, Gibco; Cat. No. 11090‐081; RRID:SCR_013551)]; 0.3‐3 mM MPP^+^ (Abcam, Cambridge, UK; Cat. No. ab144783; RRID:AB_2893161), 25 μM ZnCl_2_ (Sigma‐Aldrich; Cat. No. Z0152), 200 μM Trolox (Sigma‐Aldrich; Cat. No. 648471), 0.5 μM TPEN (Sigma‐Aldrich; Cat. No. 616394), 100 μM leupeptin (Abcam; Cat. No. ab141404), 20 μM CA‐074 (MyBioSource, San Diego, CA, USA; Cat. No. MBS696236), 100 μM zVAD (Merck Millipore; Cat. No. 627610), or 10 μM MitoTEMPO (GlpBio, Montclair, CA, USA; Cat. No. GC44206) was used.

### 2.6 Cell Death Assay

For the detection of MPP^+^‐induced cell death, propidium iodide (PI, Sigma‐Aldrich; Cat. No. P4170; RRID:AB_10794145) staining was performed. Specifically, 2.5 μg/mL PI dye was added to the medium at 22 h after treatment with MPP^+^, followed by a 15‐min incubation at room temperature. The cultures were then washed with fresh MEM to remove excessive PI dye. PI‐positive cells were analyzed using an inverted fluorescence microscope (EVOS Cell Imaging System, Thermo Fisher Scientific; RRID:SCR_019228) with excitation and emission wavelengths of 540/608 nm. To count the total number of cells, Hoechst 33258 dye (Invitrogen, Thermo Fisher Scientific, Waltham, MA, USA; Cat. No. H1398; RRID:AB_10626776) was applied at a concentration of 4 μg/mL with excitation and emission wavelengths of 352/461 nm. Cell death was further quantified by measuring the activity of lactate dehydrogenase (LDH) released into the medium from damaged cells. LDH values were normalized by scaling the sham‐wash condition to 0% and treatment with 100 μM NMDA (Abcam; Cat. No. ab120052) to 100% in sister cultures (Koh and Choi 1987).

### 2.7 Zinpyr‐1 or FluoZin‐3 AM Staining

To assess intracellular zinc levels, Zinpyr‐1 or FluoZin‐3 AM dye was used. For Zinpyr‐1 staining, cultures were preloaded with 5 μM Zinpyr‐1 (Cayman Chemical, Ann Arbor, MI, USA; Cat. No. 15122) at 37°C for 30 min prior to drug treatment. For FluoZin‐3 AM staining, 5 μM FluoZin‐3 AM (Invitrogen; Cat. No. F24195) was applied at 37°C for 30 min, 30 min before microscopic observation following drug treatment. Microscopic images were acquired using a fluorescence microscope (EVOS Cell Imaging System) with excitation and emission wavelengths of 492/527 nm for Zinpyr‐1 and 494/516 nm for FluoZin‐3. Quantification of Zinpyr‐1 and FluoZin‐3 AM fluorescence was performed by measuring fluorescence intensity within defined microscopic fields using ImageJ software.

### 2.8 Lysotracker Red Staining

For LysoTracker staining, cultures were incubated with 75 nM LysoTracker Red fluorescence dye (Invitrogen; Cat. No. L7518; RRID:AB_2801833) at 37°C for 30 min. The culture medium was then replaced with fresh MEM, followed by treatment with MPP^+^ and other chemicals based on experimental conditions. Microscopic images were obtained using a fluorescence microscope (EVOS Cell Imaging System) with excitation and emission wavelengths of 577/590 nm. Quantification of the LysoTracker signal was performed by measuring fluorescence intensity within microscopic fields using ImageJ software.

### 2.9 H_2_DCFDA Staining for Detection of the Intracellular Level of ROS

Cultures were exposed to 10 μM H_2_DCFDA fluorescence dye (ThermoFisher Scientific; Cat. No. D399; RRID:AB_2921232) at 37°C for 30 min, followed by fixation in 4% paraformaldehyde at room temperature for 15 min. Confocal microscopic images were acquired using a Leica TCS SP5 confocal microscope (Leica Microsystems, Wetzlar, Germany; RRID:SCR_018951) with excitation and emission wavelengths of 495/527 nm. Quantification of the DCF signal was performed by measuring fluorescence intensity within microscopic fields using ImageJ software.

### 2.10 Lipid Peroxidation Probe Staining

The culture medium was replaced with fresh MEM, followed by treatment with MPP^+^ and other chemicals based on experimental conditions. For lipid peroxidation staining, cultures were incubated with a 1:2000 dilution of BDP 581/591 C11 (Dojindo Molecular Technologies, Kumamoto, Japan; Cat. No. L267) at 37°C for 30 min. Microscopic images were acquired using a fluorescence microscope (EVOS Cell Imaging System) with excitation/emission wavelengths of 561/600–630 nm (red) and 488/510–550 nm (green) for BDP 581/591 C11. Quantification of BDP 581/591 C11 fluorescence was performed by measuring fluorescence intensity within microscopic fields using ImageJ software.

### 2.11 Western Blots

The cytosolic fraction of cell lysates was obtained using cytosol extraction buffer containing 250 mM Sucrose (Duksan Pure Chemicals, Ansan, South Korea; Cat. No. 848), 20 mM HEPES (Taeshin Bioscience, Seoul, South Korea; Cat. No. H3375), 10 mM KCl (Sigma‐Aldrich; Cat. No. P9333), 1.5 mM MgCl_2_ (Sigma‐Aldrich; Cat. No. M8266), 2 mM EDTA (Sigma‐Aldrich; Cat. No. EDS), and 25 μg/mL digitonin (Sigma‐Aldrich; Cat. No. D141). Protein precipitation was carried out by adding four volumes of acetone to the cytosolic extracts, followed by incubation at −20°C overnight. Pellets were collected by centrifugation at 1910 × *g* for 20 min at 4°C and resuspended in cytosol buffer containing 20 mM Tris (Duchefa Biochemie, Haarlem, Netherlands; Cat. No. T1501, pH 7.4), 150 mM NaCl (Junsei Chemical Co. Ltd., Tokyo, Japan; Cat. No. 19015S1250), 1% Triton X‐100 (Thermo Scientific Chemicals, Waltham, MA, USA; Cat. No. A16046‐AP), and 2 mM EDTA supplemented with freshly prepared protease/phosphatase inhibitors (2 μg/mL Aprotinin (Cayman Chemical; Cat. No. 14716), 2 μg/mL leupeptin (Abcam), 1 μg/mL Pepstatin A (GlpBio; Cat. No. GC11974), 1 mM PMSF (Amresco, Solon, OH, USA; Cat. No. 0754), 1 mM Na_3_VO_4_ (Sigma‐Aldrich; Cat. No. S6508), 5 mM NaF (Sigma‐Aldrich; Cat. No. S7920), and 10 mM Na_4_P_2_O_7_ (Sigma‐Aldrich; Cat. No. S6422)). Protein concentration in the cytosolic fraction was determined using a bicinchoninic acid (BCA) protein assay (Thermo Fisher Scientific; Cat. No. 23225) according to the manufacturer's instructions. Equal amounts of protein, based on these measurements, were loaded for subsequent SDS‐PAGE and western blot analyses. Separation of 20 μg of total protein was performed using SDS‐PAGE (12%) under reducing conditions with SDS (DAEJUNG Chemicals & Metals Co. Ltd., Siheung, South Korea; Cat. No. 7592‐1405), followed by transfer to a PVDF membrane. After blocking with 3% BSA solution (1% TBST, 3% BSA, and sodium azide), membranes were incubated overnight at 4°C with primary antibodies against Cathepsin B (Cell Signaling Technology, Danvers, MA, USA; Cat. No. 31718; RRID:AB_2798936) and Actin (Abbkine Scientific, Wuhan, China; Cat. No. ABL1011) diluted in 2% BSA‐containing TBST. Membranes were then washed four times with TBST and incubated with the appropriate secondary antibody for 1 h at room temperature. After additional TBST washes, immunoblot signals were visualized using an enhanced chemiluminescence (ECL) system and captured with the Fusion Solo Imaging System (Vilber Lourmat, France). Actin was used as a loading control, and ImageJ software was employed to quantify normalized band intensities.

### 2.12 Immunocytochemistry

For immunocytochemistry, differentiated midbrain dopaminergic (mDA) neurons were fixed in 4% paraformaldehyde (PFA, Dreamcell, Korea; Cat. No. P1031‐2) and subsequently washed with PBS (WellGene). Fixed cells were permeabilized in PBS containing 0.1% Triton X‐100 (Thermo Scientific Chemicals) and 0.5% BSA, which also served as the blocking solution. Cells were then incubated with primary antibodies diluted in the same permeabilization/blocking buffer. The primary antibody was used: anti‐TH (1:500; Novus Biologicals, Cat. No. NB300‐109, RRID:AB_10077691). Primary antibody incubation was performed overnight at 4°C. After three washes with PBS, cells were incubated for 1 h at room temperature with the appropriate Alexa Fluor–conjugated secondary antibodies, including Alexa Fluor 568 goat anti‐rabbit IgG (Invitrogen, Cat. No. A11011, RRID:AB_143157) and Alexa Fluor 488 goat anti‐mouse IgG (Invitrogen, Cat. No. A11001, RRID:AB_2534069). Images were acquired using a fluorescence microscope under identical exposure settings across experimental groups.

### 2.13 JC‐1 Staining for Evaluation of Change of Mitochondrial Membrane Potential

Cultures were pre‐exposed to 5 μM JC‐1 (Thermo Fisher Scientific; Cat. No. T3168; RRID:AB_10679382) at 37°C for 30 min and washed with fresh MEM. Chemical treatments were then applied according to experimental conditions. Fluorescence signals were measured using a fluorescence spectrometer (Molecular Devices, San Jose, CA, USA; RRID:SCR_018951) with excitation wavelengths of 480 and 544 nm and emission wavelengths of 545 and 575 nm.

### 2.14 MitoSOX Staining

The culture medium was replaced with fresh MEM, followed by treatment with MPP^+^ and other chemicals according to the experimental conditions. After treatment, cultures were incubated with 1 μM MitoSOX (Invitrogen; Cat. No. M36008) at 37°C for 30 min. For microscopic imaging, a fluorescence microscope (EVOS Cell Imaging System) was used with excitation and emission wavelengths of 396 nm and 580–610 nm, respectively. Quantification of MitoSOX fluorescence was performed by measuring the fluorescence intensity within microscopic fields using ImageJ software.

### 2.15 Reverse‐Transcription Polymerase Chain Reaction (RT‐PCR)

Total RNA was isolated from Rho^0^ CHO cells using Trizol reagent (Invitrogen; Cat. No. 15596‐018; RRID:AB_2707448). One microgram of RNA was reverse transcribed to cDNA using the iScript^TM^ cDNA synthesis kit (Bio‐Rad Laboratories, Hercules, CA, USA; Cat. No. 1708890; RRID:SCR_019195). PCR amplification was performed using AccuPower PCR PreMix (BIONEER, Daejeon, South Korea; Cat. No. K‐2012). Primers for mtDNA (forward: 5′‐CCG GCG TAA AAC GTG TTA TAG ACT‐3′, reverse: 5′‐GTA TTA GGT ATA ATA TCG GCA GTC‐3′) and GAPDH (forward: 5′‐CAA AGG CAC AGT CAA GGC TGA‐3′, reverse: 5′‐TGG TGA AGA CGC CAG TAG ATT‐3′) were used. PCR products were separated on 2% agarose gels (Cellnest, iCellSci, South Korea; Cat. No. CNA004‐0500), stained with RedSafe Nucleic Acid Staining Solution (Intron Biotechnology, Seongnam, South Korea; Cat. No. 21141) and visualized using the MF‐ChemiBIS imaging system (DNR Bio‐Imaging Systems, Israel; Cat. No. 760‐1‐32).

### 2.16 Quantitative Image Analysis

mDA images were analyzed using ImageJ (NIH). All images were first converted to 8‐bit grayscale, and background signals were reduced using the background subtraction tool. A uniform thresholding strategy was applied across all samples within each experiment to ensure consistent detection parameters. Following thresholding, signals were quantified using the *Analyze Particles* function. All images were processed using identical analysis settings.

### 2.17 Statistical Analysis

Statistical analyses were performed using GraphPad Prism (version 9.10). Data are presented as mean ± SEM. Details of all statistical tests, including exact *t* values, degrees of freedom, *F* values, and exact *p* values for all experiments, are provided in the Appendix S1. Two‐tailed Student's *t*‐tests were used for single comparisons, and one‐way ANOVA followed by Dunnett's or Tukey's post hoc tests was applied for multiple‐group comparisons, as indicated in the figure legends. Dunnett's test was used when comparing each treatment group with a single control group, whereas Tukey's test was applied for pairwise comparisons among multiple treatment groups. Quantitative data were normalized to the control group and expressed as percentages or fold changes, as specified in each figure. These normalization procedures did not artificially constrain variance. No formal outlier tests were performed, and no data points were excluded. Normality of data distribution was not formally assessed because primary astrocyte and neuronal culture experiments typically involve small sample sizes (3–5 biological replicates), for which tests of normality have limited reliability. Under these conditions, parametric tests such as *t*‐tests and ANOVA are widely accepted and considered robust; accordingly, we followed established practice in the field (McCarthy and de Vellis, 1980; Schildge et al. 2013; Aizenman et al. 2000; Sensi, Ton‐That, Sullivan, et al. 2003; Settembre et al. 2013; Jiang and Mizushima 2013). No statistical methods were used to predetermine sample sizes; instead, sample sizes were determined based on established practice in similar primary culture studies and prior work on zinc‐induced neurotoxicity, mitochondrial dysfunction, and lysosomal/autophagy regulation. A *p* value less than 0.05 was considered statistically significant. Statistical significance is indicated as follows: ns, *p* > 0.05; *p < 0.05; **p < 0.01; ***p < 0.001. No blinding was performed during experimentation or data analysis.

## 3 Result

### 3.1 Mediation by Zinc of MPP ^+^‐Induced Neuronal Death

**FIGURE 1:**
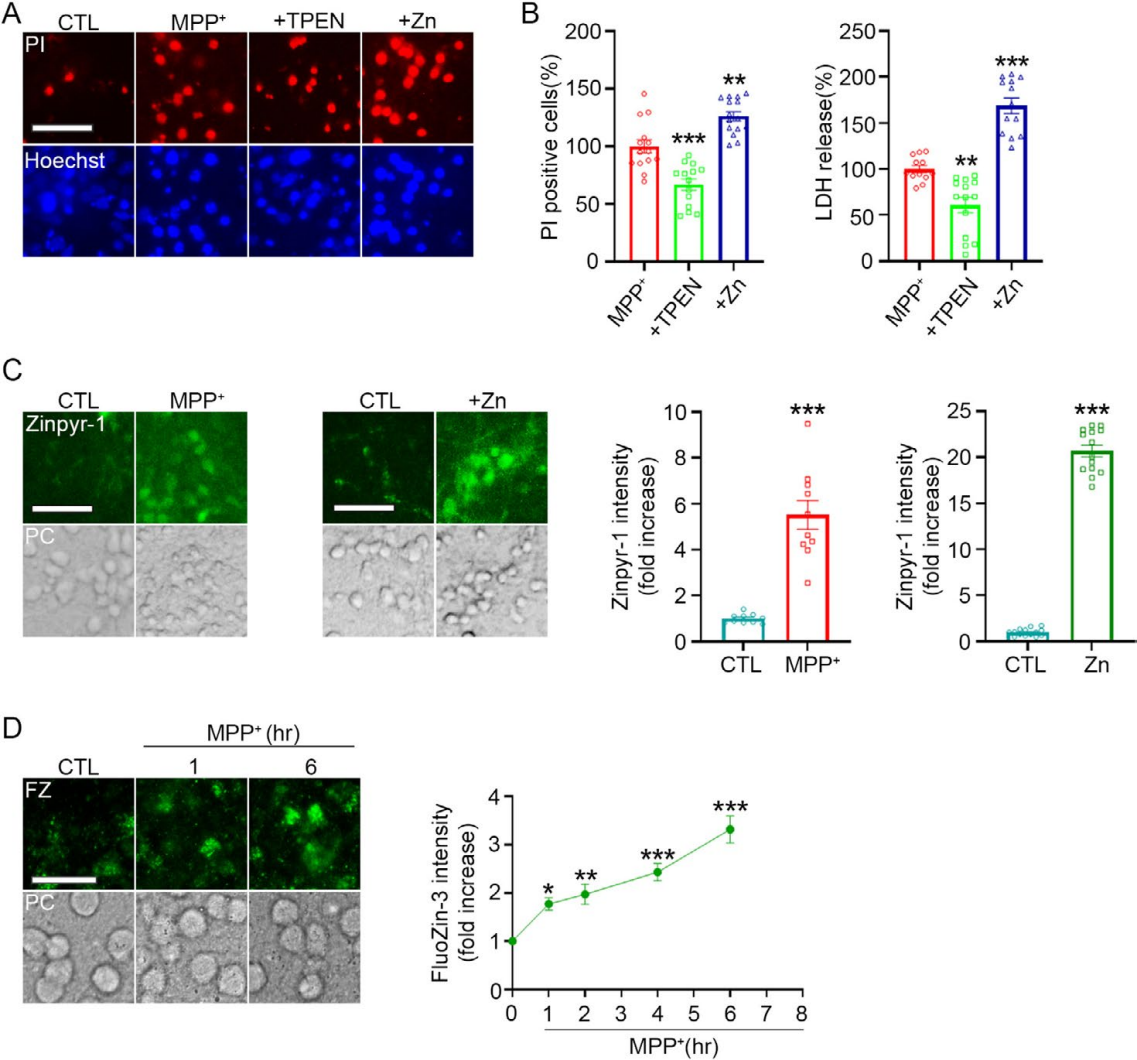
Cytosolic zinc mediates MPP^+^‐induced neuronal death. (A) Representative fluorescence images of propidium iodide (PI)‐positive dead cells (top) and Hoechst 33258‐stained nuclei (bottom) in mouse cerebrocortical cultures 22 h after treatment with 300 μM MPP^+^, with or without 0.5 μM TPEN or 25 μM ZnCl_2_. Scale bar, 50 μm. (B) Quantification of PI‐positive cells (left) and LDH release (right) following MPP^+^ treatment, with or without TPEN or ZnCl_2_. Data are shown as mean ± SEM. For PI‐positive cells, *n* = 14–15 measurements per condition, obtained from 3 to 5 independent cultures (biological replicates), with 3–5 different treatment groups per culture (technical replicates). For LDH release, *n* = 12–14 per condition, obtained from 3 to 5 independent cultures with multiple replicates. For PI‐positive cells, *F* (2, 40) = 37.10, *p* < 0.0001; for LDH release, *F* (2, 36) = 57.26, *p* < 0.0001. Statistical analysis was performed using one‐way ANOVA followed by Dunnett's post hoc test. (C) Fluorescence images (left) and quantification (right) of Zinpyr‐1 after 14 h exposure to MPP^+^ or ZnCl_2_. Zinpyr‐1 fluorescence intensity was quantified using ImageJ (mean ± SEM). *n* = 10–14 per condition, from 3 to 5 independent cultures with 3–5 microscopic fields per culture. For MPP^+^ treatment, *t*(18) = 7.14, *p* < 0.0001; for ZnCl_2_ treatment, *t*(26) = 30.85, *p* < 0.0001. Statistical analysis was performed using two‐tailed Student's *t*‐test. Scale bar, 50 μm. (D) Fluorescence images (left) and quantification (right) of FluoZin‐3 staining in cerebrocortical cultures exposed to 300 μM MPP^+^ for the indicated time points. FluoZin‐3 intensity was measured using ImageJ (mean ± SEM). *n* = 5–6 per time point, from 3 independent cultures, with multiple technical replicates per culture. *F* (4, 21) = 19.89, *p* < 0.0001. Statistical analysis was performed using one‐way ANOVA followed by Dunnett's post hoc test. Scale bar, 20 μm.

Previous research by Sheline et al. has demonstrated that an increase in intracellular zinc levels and a decrease in the NAD^+^/NADH ratio mediate neuronal death induced by mitochondrial complex I and II inhibitors (Sheline et al. 2013). In this study, we confirmed that zinc is involved in MPP^+^‐induced cell death in mouse cerebrocortical cultures. Chelation of zinc with TPEN (N,N,N′,N′‐tetrakis(2‐pyridinylmethyl)‐1,2‐ethanediamine) significantly attenuated MPP^+^‐induced neuronal death, whereas the addition of exogenous zinc exacerbated it (Figure 1A,B), suggesting a crucial role for zinc in MPP^+^‐induced neuronal death. Moreover, we observed a significant increase in free intracellular zinc levels starting at 14 h after MPP^+^ exposure, as detected by the Zinpyr‐1 fluorescence probe (Figure 1C). To further determine the earliest time point at which cytosolic zinc elevation occurs, we performed fluorescence imaging using FluoZin‐3. This analysis revealed that intracellular zinc levels increased to a significant extent as early as 30 min following MPP^+^ treatment (Figure 1D).

### 3.2 ROS‐Dependent Increase in Intracellular Zinc by MPP ^+^

**FIGURE 2:**
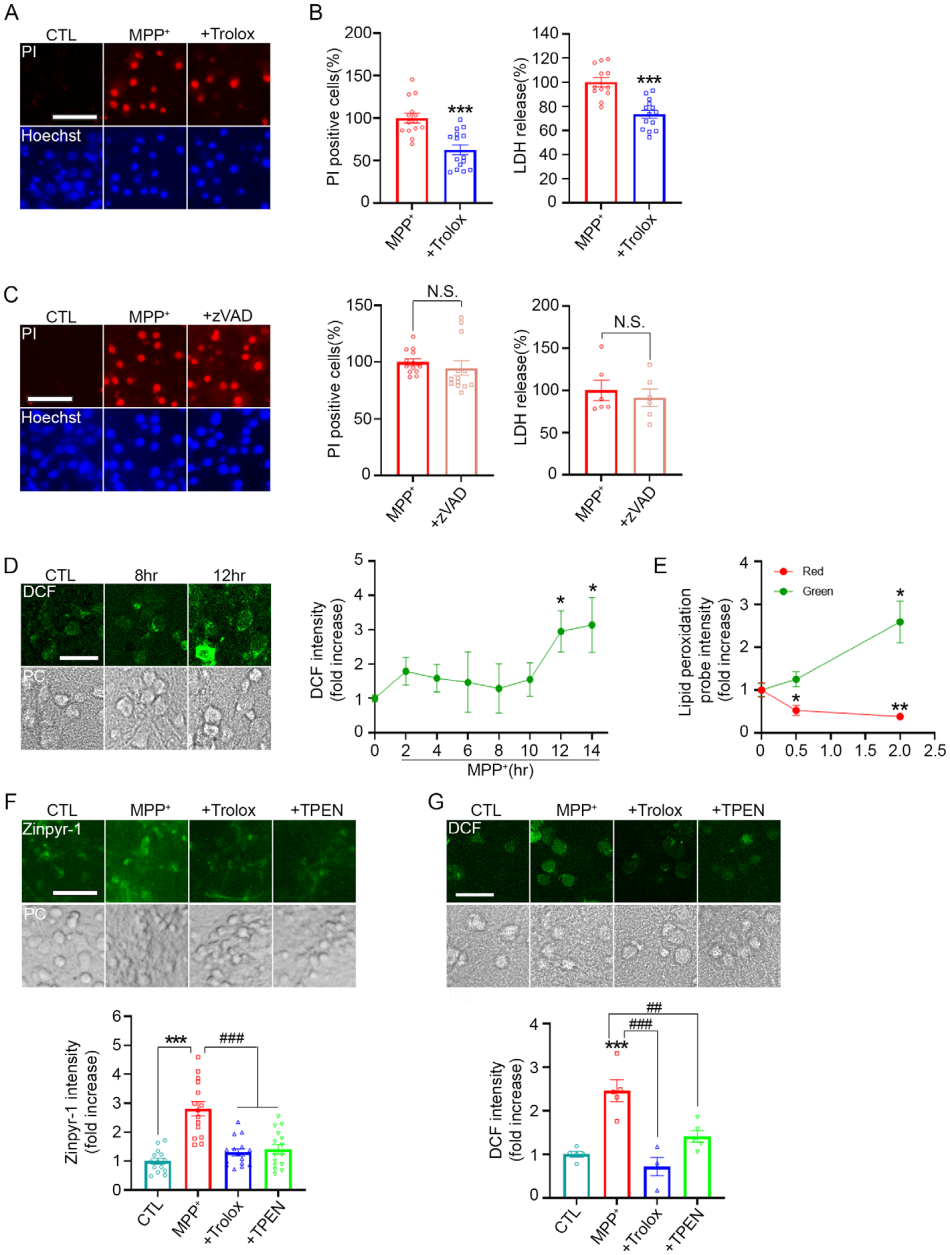
MPP^+^‐induced ROS production promotes intracellular zinc accumulation. (A) Representative fluorescence images of propidium iodide (PI)‐positive dead cells (top) and Hoechst 33258‐stained nuclei (bottom) in mouse cerebrocortical cultures 22 h after treatment with 300 μM MPP^+^, with or without 200 μM Trolox. Scale bar, 50 μm. (B) Quantification of PI‐positive cells (left) and LDH release (right) in cultures treated as in (A). Data are presented as mean ± SEM. *n* = 14–15 per condition for PI‐positive cells and 12–14 per condition for LDH release, obtained from 3 to 5 independent cultures (biological replicates), with 3–5 technical replicates per culture. For PI‐positive cells, *t*(27) = 4.59, *p* < 0.0001; for LDH release, *t*(24) = 5.27, *p* < 0.0001. Statistical analysis was performed using two‐tailed Student's *t*‐test. (C) Fluorescence images (left) and quantification of PI‐positive cells or LDH release (right) in mouse cerebrocortical cultures 22 h after treatment with 300 μM MPP^+^, with or without 100 μM zVAD. Data are presented as mean ± SEM. *n* = 13 per condition for PI‐positive cells and *n* = 6 per condition for LDH release, from 3 independent cultures, with multiple technical replicates per culture. For PI‐positive cells, *t*(24) = 0.75, *p* = 0.4580; for LDH release, *t*(10) = 0.53, *p* = 0.6059. Statistical analysis was performed using two‐tailed Student's *t*‐test. Scale bar, 50 μm. (D) Confocal fluorescence images (left) and quantification (right) of DCF staining in cerebrocortical cultures exposed to 300 μM MPP^+^ for the indicated time points. DCF intensity was quantified using ImageJ (mean ± SEM). *n* = 6–15 per time point, obtained from three independent cultures with multiple technical replicates per culture. *F* (7, 78) = 1.98, *p* = 0.0690. Statistical analysis was performed using one‐way ANOVA followed by Dunnett's post hoc test. (E) Quantification of Lipid peroxidation probe staining in cultures exposed to 300 μM MPP^+^ for the indicated time points. Lipid peroxidation intensity was measured using ImageJ (mean ± SEM). Red channel: *n* = 8 per condition; green channel: *n* = 4–6 per condition, from 3 independent cultures with multiple technical replicates. For red channel, *F*(2, 21) = 6.94, *p* = 0.0049; for green channel, *F* (2, 13) = 6.25, *p* = 0.0125. Statistical analysis was performed using one‐way ANOVA followed by Dunnett's post hoc test. (F) Fluorescence images (top) and quantification (bottom) of Zinpyr‐1 fluorescence in cultures treated with MPP^+^ for 12 h, with or without 200 μM Trolox or 0.5 μM TPEN. Zinpyr‐1 intensity was quantified using ImageJ (mean ± SEM). *n* = 14–15 per condition, from 3 to 4 independent cultures, with multiple technical replicates per culture. *F*(3, 55) = 21.92, *p* < 0.0001. Statistical analysis was performed using one‐way ANOVA followed by Tukey's post hoc test. Scale bar, 50 μm. (G) Confocal fluorescence images (top) and quantification (bottom) of DCF fluorescence in cultures treated with MPP^+^ for 12 h, with or without Trolox or TPEN. DCF intensity was quantified using ImageJ (mean ± SEM). *n* = 4–5 per condition, from 3 independent cultures with multiple technical replicates per culture. *F* (3, 15) = 18.50, *p* < 0.0001. Statistical analysis was performed using one‐way ANOVA followed by Tukey's post hoc test. Scale bar, 25 μm.

Multiple studies have shown that MPP^+^ induces ROS production and triggers the release of intracellular zinc (Lee et al. 2009; Rojas et al. 2005; Sheline et al. 2013). In this study, we aimed to determine whether ROS mediates the MPP^+^‐induced increase in intracellular zinc levels. We first observed that Trolox (6‐hydroxy‐2,5,7,8‐tetramethylchroman‐2‐carboxylic acid), an antioxidant vitamin E analog, significantly reduced MPP^+^‐induced neuronal death (Figure 2A,B), indicating a critical role for ROS in MPP^+^‐mediated neurotoxicity. In contrast, treatment with the caspase inhibitor zVAD failed to reduce cell death, demonstrating that MPP^+^‐induced neurotoxicity in mouse cortical cultures does not occur through an apoptotic mechanism (Figure 2C).

Consistently, a significant increase in ROS was detected from 12 h after MPP^+^ treatment, as measured by DCF fluorescence in cerebrocortical cultures (Figure 2D). To complement the DCF assay with a quantitative measure of lipid peroxidation, we performed staining using the BDP 581/591 C11 lipid peroxidation probe. This probe emits red fluorescence in its unoxidized state and shifts to green fluorescence upon reacting with lipid radicals generated during lipid peroxidation. Following MPP^+^ exposure, red fluorescence decreased significantly as early as 0.5 h, and by 2 h both a robust reduction in red fluorescence and a marked increase in green fluorescence were observed. These results indicate that ROS generation occurs rapidly, beginning within 30 min of MPP^+^ treatment (Figure 2E).

To examine whether ROS mediates the increase in intracellular zinc, we measured intracellular free zinc concentration ([Zn^2+^]_i_) following treatment with either Trolox or TPEN. Both treatments almost completely restored [Zn^2+^]_i_ to control levels (Figure 2F), suggesting that ROS is a major upstream driver of zinc elevation. We next investigated whether the zinc, in turn, affects ROS production. Trolox reduced ROS levels below basal values, while TPEN led to only a slight but statistically significant reduction in MPP^+^‐induced ROS (Figure 2G).

Taken together, these findings indicate that ROS generation precedes and mediates the increase in [Zn^2+^]_i_ following MPP^+^ exposure, and that elevated cytosolic zinc may further enhance ROS production, forming a feedforward loop that contributes to neuronal damage.

### 3.3 A Critical Role of Lysosomal Membrane Permeabilization in MPP ^+^‐Mediated Neuronal Death

When intracellular free zinc levels rise, zinc is typically buffered by zinc‐binding proteins such as metallothionein, exported to the extracellular space via ion channels or transporters, or sequestered into intracellular organelles to maintain cytosolic zinc homeostasis (Takeda 2010; Kambe 2011). However, a rapid increase in cytosolic free zinc can lead to abnormal lysosomal membrane permeabilization (LMP), allowing zinc to enter lysosomes and destabilize their membranes (Hwang et al. 2008; Dehay et al. 2010). This process results in the release of lysosomal proteases, including cathepsin B (CTSB), into the cytoplasm, ultimately promoting cell death (Kim et al. 2020; Koh et al. 2019). In animal models treated with MPTP, LMP has been shown to mediate neuronal death by facilitating the cytosolic leakage of lysosomal proteolytic enzymes. Additionally, LMP contributes to lysosomal deficiency, which impairs autophagic flux and leads to the accumulation of protein aggregates (Dehay et al. 2010).

**FIGURE 3:**
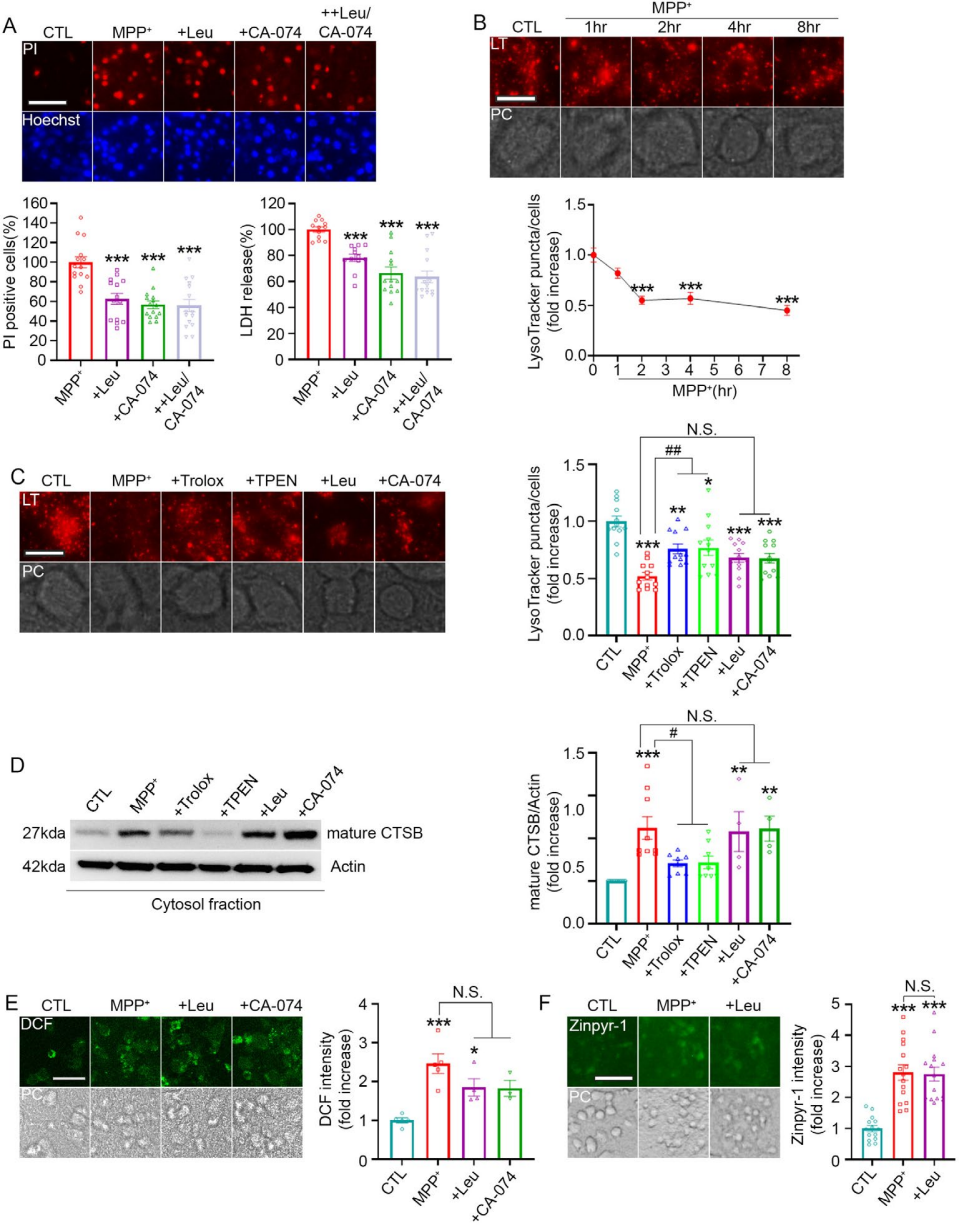
MPP^+^‐induced ROS and zinc contribute to LMP. (A) Representative fluorescence images (top) of propidium iodide (PI)‐positive dead cells (red) and quantification (bottom) of PI‐positive cell counts and LDH release in mouse cortical cultures treated with 300 μM MPP^+^ for 22 h, with or without 100 μM leupeptin or 20 μM CA‐074. PI‐positive cells were quantified using ImageJ, and LDH release was expressed as relative values. Data are presented as mean ± SEM. *n* = 14–15 per condition for PI‐positive cells and *n* = 12–14 per condition for LDH release, obtained from 3 to 5 independent cultures (biological replicates), with 3–5 technical replicates per culture. For PI‐positive cells, *F* (3, 54) = 14.72, *p* < 0.0001; for LDH release, *F*(3, 47) = 18.31, *p* < 0.0001. Statistical analysis was performed using one‐way ANOVA followed by Dunnett's post hoc test. Scale bar, 50 μm. (B) Fluorescence images (top) and quantification (bottom) of LysoTracker fluorescence in cultures exposed to 300 μM MPP^+^ for the indicated time points. LysoTracker intensity was quantified using ImageJ. Data are presented as mean ± SEM. *n* = 14 per condition, obtained from 3 to 4 independent cultures, with 3–5 technical replicates per culture. *F* (4, 65) = 16.16, *p* < 0.0001. Statistical analysis was performed using one‐way ANOVA followed by Dunnett's post hoc test. Scale bar, 10 μm. (C) Fluorescence images (left) and quantification (right) of LysoTracker fluorescence in cultures treated with 300 μM MPP^+^ for 4 h with or without 200 μM Trolox, 0.5 μM TPEN, 100 μM leupeptin, or 20 μM CA‐074. LysoTracker intensity was analyzed using ImageJ. Data are presented as mean ± SEM. *n* = 12 per condition, obtained from 3 to 5 independent cultures, with 3–5 technical replicates per culture. *F* (5, 66) = 11.34, *p* < 0.0001. Statistical analysis was performed using one‐way ANOVA followed by Tukey's post hoc test. Scale bar, 10 μm. (D) Western blot analysis (left) and quantification (right) of cytosolic cathepsin B in mouse cortical cultures treated with 300 μM MPP^+^ for 10 h, with or without 200 μM Trolox, 0.5 μM TPEN, 100 μM leupeptin, or 20 μM CA‐074. Actin was used as a loading control. Quantification of cathepsin B level in the cytoplasm was analyzed using ImageJ. Data are presented as mean ± SEM. *n* = 4–10 per condition, obtained from 3 to 5 independent cultures, with multiple technical replicates per culture. *F* (5, 37) = 7.55, *p* < 0.0001. Statistical analysis was performed using one‐way ANOVA followed by Tukey's post hoc test. (E) Confocal images (left) and quantification (right) of DCF fluorescence in mouse cortical cultures treated with MPP^+^ for 12 h, with or without 100 μM leupeptin or 20 μM CA‐074. DCF intensity was quantified using ImageJ. Data are presented as mean ± SEM. *n* = 3–5 per condition, obtained from 3 to 5 independent cultures, with 3–5 technical replicates per culture. *F* (3, 13) = 10.59, *p* = 0.0008. Statistical analysis was performed using one‐way ANOVA followed by Tukey's post hoc test. Scale bar, 10 μm. (F) Fluorescence images (left) and quantification (right) of Zinpyr‐1 fluorescence in cultures treated with MPP^+^ for 12 h, with or without 100 μM leupeptin. Zinpyr‐1 intensity was measured using ImageJ. Data are presented as mean ± SEM. *n* = 14–15 per condition, obtained from 3 to 5 independent cultures, with 3–5 technical replicates per culture. *F* (2, 41) = 24.12, *p* < 0.0001. Statistical analysis was performed using one‐way ANOVA followed by Tukey's post hoc test. Scale bar, 50 μm.

In this study, we investigated whether MPP^+^ induces LMP in primary cortical cultures and whether zinc acts as a critical mediator in this process. Treatment with lysosomal protease inhibitors, leupeptin or CA‐074, significantly reduced MPP^+^‐induced neuronal death (Figure 3A), suggesting a role for lysosomal enzymes in MPP^+^ toxicity. To assess LMP and lysosomal integrity, we utilized LysoTracker Red, a dye that fluoresces in acidic compartments. Following MPP^+^ treatment, LysoTracker fluorescence was markedly reduced, indicating rapid LMP and lysosomal loss (Figure 3B). This reduction was significantly restored by Trolox or TPEN, but not by leupeptin or CA‐074 (Figure 3C), suggesting that lysosomal protease inhibitors act downstream of LMP, possibly by blocking CTSB activity after its cytosolic release.

To directly examine whether MPP^+^ induces cytosolic leakage of cathepsin B, we performed Western blot analysis using cytosolic fractions obtained through digitonin‐based selective permeabilization, which preserves intracellular organelles. Under these conditions, cathepsin B—normally restricted to lysosomes—was clearly detected in the cytosol following MPP^+^ treatment. Importantly, Trolox and TPEN markedly reduced this MPP^+^‐induced cytosolic release of cathepsin B, whereas leupeptin and CA‐074 did not inhibit its leakage (Figure 3D). These results further demonstrate that MPP^+^‐induced ROS elevation and zinc accumulation act upstream of LMP.

We next examined whether lysosomal protease inhibitors affect MPP^+^‐induced ROS production and the increase in [Zn^2+^]_i_. Neither leupeptin nor CA‐074 altered the MPP^+^‐induced elevation of ROS, as assessed by DCF fluorescence (Figure 3E). Similarly, MPP^+^‐induced increases in [Zn^2+^]_i_ visualized using Zinpyr‐1, were not affected by leupeptin (Figure 3E). Notably, treatment with 20 μM CA‐074 alone produced a strong green fluorescence with Zinpyr‐1 (data not shown), precluding accurate interpretation; therefore, only the effect of leupeptin is presented (Figure 3F).

Taken together, our findings suggest that MPP^+^‐induced ROS and cytosolic zinc elevations act upstream of LMP, leading to the release of CTSB into the cytoplasm and contributing to neuronal death.

### 3.4 Zinc‐Mediated MPP ^+^ Neurotoxicity in Midbrain Dopaminergic Neurons Derived From Human iPSCs

**FIGURE 4:**
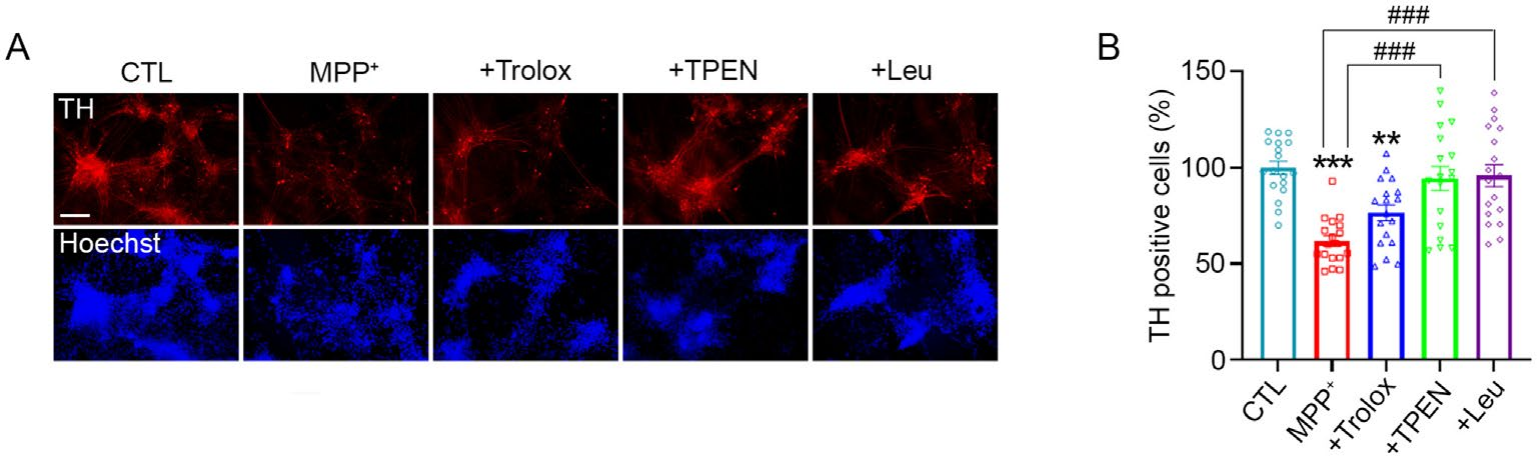
Neuroprotective effects of drugs against MPP^+^‐mediated reduction of tyrosine hydroxylase (TH)‐positive neurons. (A) Representative fluorescence images of TH‐positive neurons and DAPI‐stained nuclei in human induced pluripotent stem cell (hiPSC)‐derived dopaminergic neuronal cultures treated with 600 μM MPP^+^ for 24 h, with or without 40 μM Trolox, 0.1 μM TPEN, or 20 μM leupeptin. Scale bar, 200 μm. (B) Quantification of TH‐positive signals per image area, normalized to DAPI using ImageJ. Data are presented as mean ± SEM. *n* = 18 per condition, obtained from 3 independent cultures, with 3 technical replicates per culture. *F* (4, 85) = 11.83, *p* < 0.0001. Statistical analysis was performed using one‐way ANOVA followed by Tukey's post hoc test.

Since dopaminergic neurons are the most vulnerable in PD, we next examined the effects of MPP^+^ in tyrosine hydroxylase (TH)‐positive dopaminergic neurons differentiated from human iPSCs. Consistent with our findings in cerebrocortical cultures, MPP^+^ markedly induced the death of TH‐positive dopaminergic neurons. Although Trolox showed only a modest trend toward reducing MPP^+^‐induced TH‐positive dopaminergic neuronal death, both TPEN and leupeptin significantly attenuated MPP^+^‐induced neurotoxicity (Figure 4). These results indicate that neuronal death induced by MPP^+^ in human iPSC‐derived midbrain dopaminergic neurons is mediated through zinc elevation and LMP, consistent with the mechanisms identified in cerebrocortical neurons.

### 3.5 MT‐3 Buffers Cytosolic Zinc and Mitigates MPP ^+^‐Induced Cytotoxicity

Metallothionein‐3 (MT‐3) is a zinc‐binding protein that regulates cytoplasmic free zinc levels. Under oxidative stress, however, MT‐3 releases its bound zinc, leading to increased cytoplasmic zinc concentration (Lee et al. 2010; Lee and Koh 2010). Koh et al. previously demonstrated that hydrogen peroxide (H_2_O_2_) treatment elevates intracellular free zinc levels in primary astrocyte cultures. This effect was absent in MT‐3 knock‐out (KO) astrocytes, where cell death was prevented (Lee et al. 2010). Among the four MT isoforms (MT‐1 to MT‐4), MT‐3 is most abundantly expressed in the central nervous system (Pedersen et al. 2012; West et al. 2008). Although both neurons and astrocytes in adult murine brains express MT‐3 (Hozumi et al. 2008; Masters et al. 1994), in primary cultures derived from fetal mouse brains, MT‐3 expression is restricted to astrocytes (Lee et al. 2010). Based on this, we utilized astrocytes from MT‐3 wild‐type (WT) and KO mice to investigate whether MT‐3 contributes to the MPP^+^‐induced increase in intracellular zinc.

**FIGURE 5:**
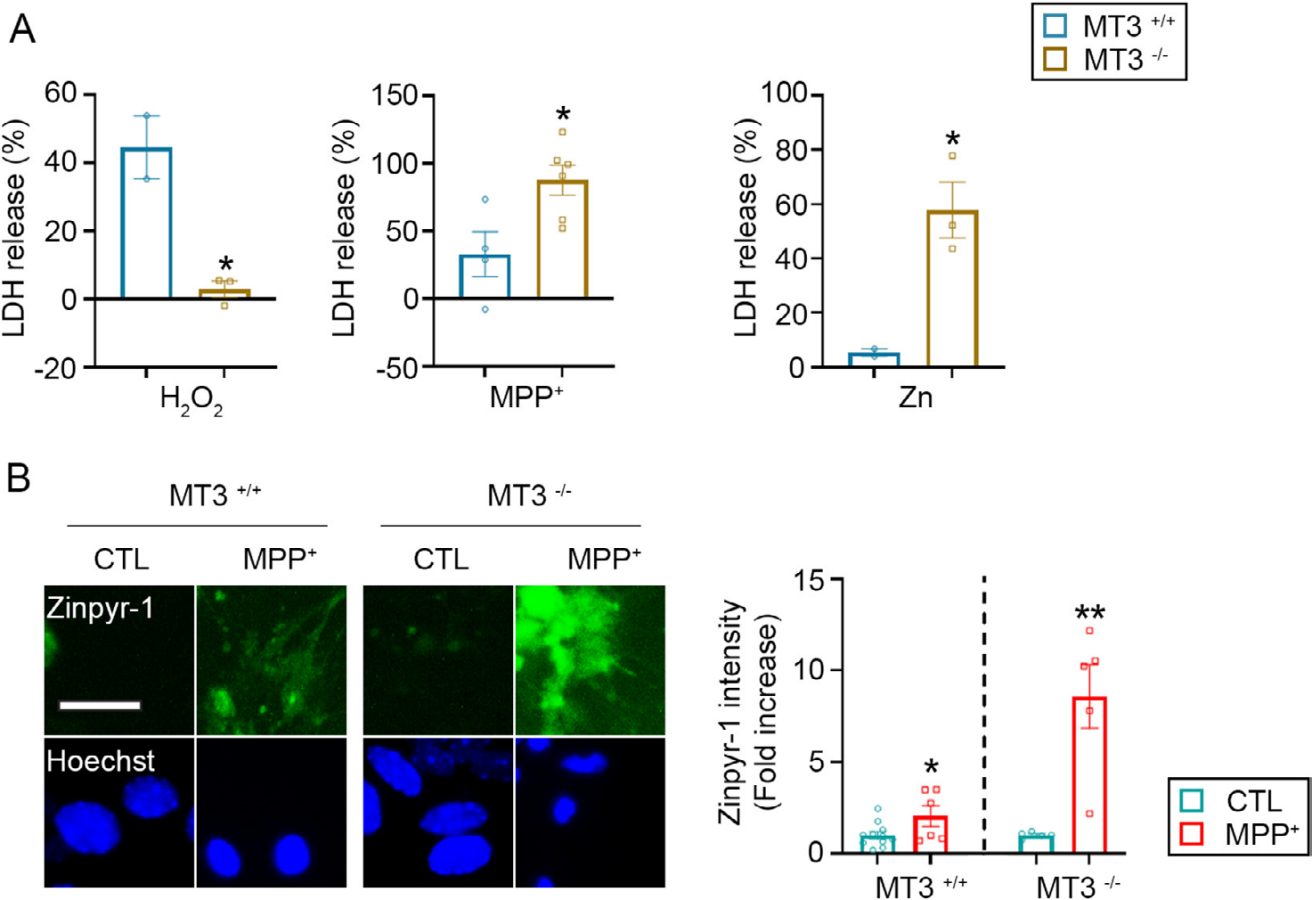
MPP^+^‐induced cell death is increased in MT3−/− astrocyte cultures. (A) LDH release measured in primary cerebrocortical astrocyte cultures derived from wild‐type (MT3+/+) or MT3 KO (MT3−/−) mice. Cultures were treated with 100 μM H_2_O_2_ or 1 mM MPP^+^ for 33 h, or with 100 μM ZnCl_2_ for 4 h. Data are presented as mean ± SEM. Sample sizes were as follows: *n* = 2–3 per condition for H_2_O_2_, *n* = 4–6 per condition for MPP^+^, and *n* = 2–3 per condition for ZnCl_2_, obtained from 3 to 5 independent cultures (biological replicates), each with 3–5 technical replicates. For H_2_O_2_, *t*(3) = 5.52, *p* = 0.0117; for MPP^+^, *t*(8) = 2.85, *p* = 0.0215; for ZnCl_2_, *t*(3) = 3.94, *p* = 0.0291. Statistical analysis was performed using two‐tailed Student's *t*‐test. (B) Representative fluorescence images (left) and quantification (right) of Zinpyr‐1 fluorescence in MT3+/+ or MT3−/− astrocyte cultures. Cultures were treated with 1 mM MPP^+^ for 30 h, and Zinpyr‐1 intensity was quantified using ImageJ. Data are presented as mean ± SEM. Sample sizes: *n* = 5–11 per condition for MT3+/+ and *n* = 5 per condition for MT3−/−, obtained from 3 to 5 independent cultures with 3–5 technical replicates per culture. For MT3+/+, *t*(15) = 2.18, *p* = 0.0459; for MT3−/−, *t*(8) = 4.34, *p* = 0.0025. Statistical analysis was performed using two‐tailed Student's *t*‐test. Scale bar, 50 μm.

Unlike the response to H_2_O_2_, MPP^+^ toxicity was significantly more severe in MT‐3 KO astrocytes (Figure 5A). This result suggests that although both H_2_O_2_ and MPP^+^ induce cell death via a shared mechanism involving elevated zinc, the source of this zinc may differ. While MT‐3 may act as a zinc donor under oxidative conditions like H_2_O_2_ exposure, it appears to play a protective role in the context of MPP^+^‐induced cytotoxicity by buffering excess zinc. To distinguish whether MT‐3 serves as a zinc source or as a zinc chelator during MPP^+^ treatment, we measured intracellular zinc levels in MT‐3 WT and KO astrocytes following MPP^+^ exposure. A significantly greater increase in cytosolic zinc was observed in MT‐3 KO cells (Figure 5B), indicating that MT‐3 helps limit free zinc accumulation and thereby confers protection. This zinc‐buffering function was further supported by the pronounced vulnerability of MT‐3 KO astrocytes to zinc overload (Figure 5A, right panel), reinforcing the role of MT‐3 as a key regulator of cytosolic zinc homeostasis.

### 3.6 Loss of Mitochondrial Membrane Potential (MMP) and Mitochondrial ROS Generation Precede Cytosolic ROS Elevation, Zinc Rise, and LMP in MPP ^+^‐Treated Neurons

In addition to MT‐3 protein, intracellular organelles such as lysosomes and mitochondria play essential roles in maintaining zinc homeostasis (Sensi, Ton‐That, Sullivan, et al. 2003; Kukic et al. 2014). Cytosolic zinc can be transiently buffered through sequestration into these organelles, whereas cellular stress may induce the release of zinc back into the cytosol, thereby elevating intracellular zinc levels (Takeda 2010; Kambe 2011). Given that MPP^+^ is a mitochondrial toxin, we investigated whether mitochondria constitute an early source of zinc dysregulation following MPP^+^ exposure.

**FIGURE 6:**
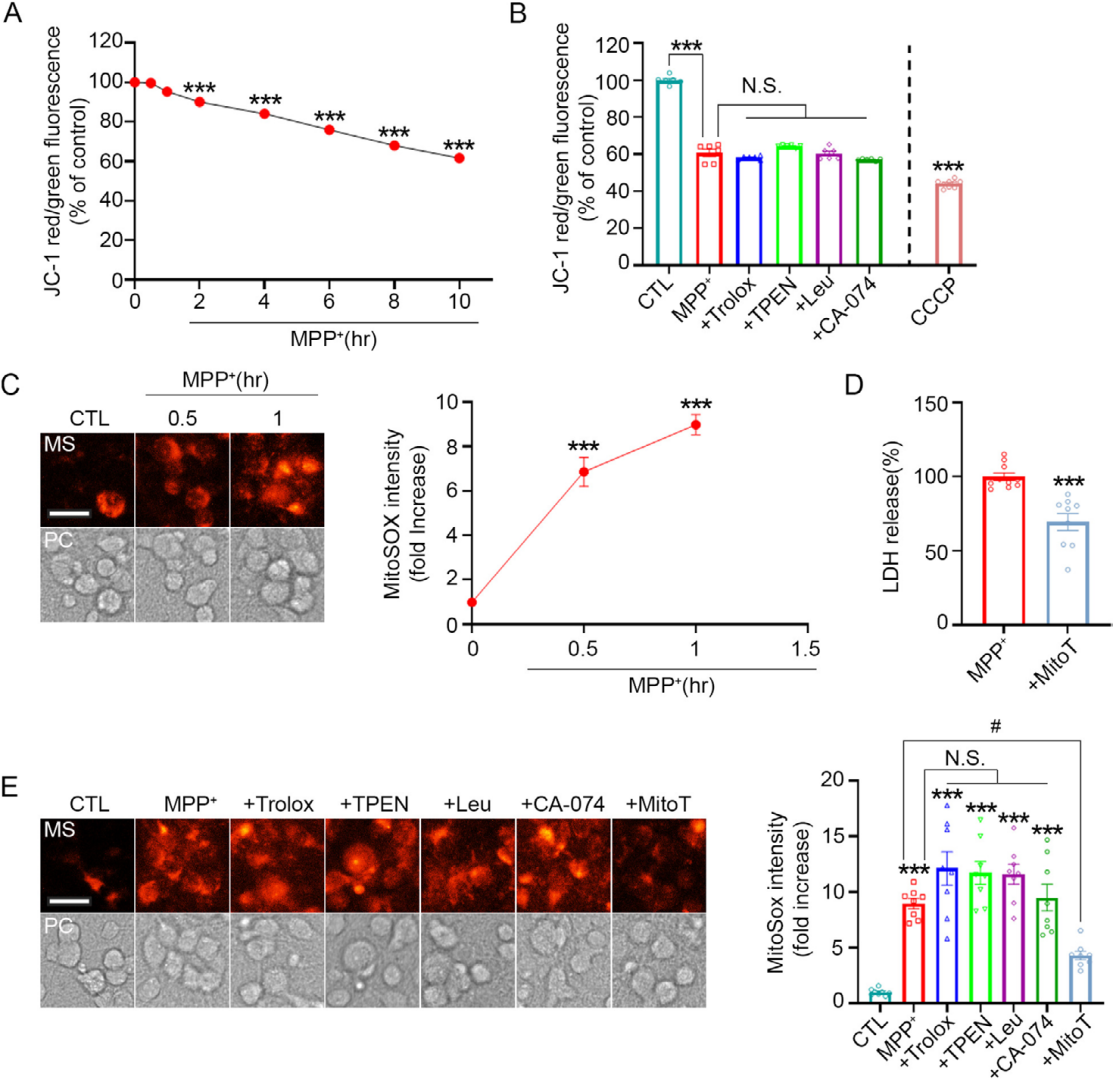
MPP^+^‐induced mitochondrial depolarization is not attenuated by antioxidant treatment or zinc chelation. (A) Quantification of JC‐1 red/green fluorescence intensity ratio in mouse cerebrocortical cultures exposed to 300 μM MPP^+^ for the indicated time points. A decrease in the red (~575 nm)/green (~545 nm) fluorescence ratio reflects mitochondrial membrane depolarization. Data are presented as mean ± SEM. *n* = 8–10 per time point, obtained from 3 independent cultures with multiple technical replicates per culture. *F* (7, 69) = 96.94, *p* < 0.0001. Statistical analysis was performed using one‐way ANOVA followed by Dunnett's post hoc test. (B) JC‐1 red/green ratio measured 6 h after sham wash or treatment with 300 μM MPP^+^, with or without Trolox, TPEN, leupeptin, or CA‐074. Treatment with 10 μM CCCP served as a positive control. Data are presented as mean ± SEM. *n* = 6 per condition, from 3 independent cultures with three technical replicates per culture. *F* (6, 38) = 318.6, *p* < 0.0001. Statistical analysis was performed using one‐way ANOVA followed by Tukey's post hoc test. (C) Representative fluorescence images (left) and quantification (right) of MitoSOX fluorescence in cultures exposed to 300 μM MPP^+^ for the indicated time points. MitoSOX intensity was quantified using ImageJ. Data are presented as mean ± SEM. *n* = 8 per time point, from 3 independent cultures with multiple technical replicates per culture. *F* (2, 21) = 77.06, *p* < 0.0001. Statistical analysis was performed using one‐way ANOVA followed by Dunnett's post hoc test. Scale bar, 25 μm. (D) LDH release measured 22 h after treatment with 300 μM MPP^+^, with or without 10 μM MitoTEMPO. Data are presented as mean ± SEM. *n* = 9–10 per condition for LDH release, from 3 independent cultures with multiple technical replicates per culture. *t*(17) = 5.01, *p* = 0.0001. Statistical analysis was performed using two‐tailed Student's *t*‐test. (E) Fluorescence images (left) and quantification (right) of MitoSOX fluorescence 1 h after sham wash or treatment with 300 μM MPP^+^, with or without Trolox, TPEN, leupeptin, or CA‐074. MitoSOX intensity was quantified using ImageJ. Data are presented as mean ± SEM. *n* = 8 per condition, from 3 independent cultures with multiple technical replicates per culture. *F* (6, 49) = 21.52, *p* < 0.0001. Statistical analysis was performed using one‐way ANOVA followed by Tukey's post hoc test. Scale bar, 25 μm.

Using JC‐1 dye to monitor mitochondrial membrane potential (MMP), we observed a significant reduction in the red/green fluorescence ratio as early as 2 h after MPP^+^ treatment, indicating mitochondrial depolarization (Figure 6A). JC‐1 accumulates in healthy mitochondria, forming red‐fluorescent aggregates, whereas in depolarized mitochondria, it remains in its monomeric green‐fluorescent form (Perelman et al. 2012). Thus, a decrease in the red/green ratio reflects MMP loss.

To determine whether MMP loss occurs upstream of zinc elevation, cytosolic ROS generation, or lysosomal membrane permeabilization (LMP), we examined whether Trolox, TPEN, leupeptin, or CA‐074 could prevent mitochondrial depolarization. None of these treatments restored MMP (Figure 6B), suggesting that mitochondrial dysfunction occurs earlier than the increase in ROS generation, intracellular zinc, or LMP. Carbonyl cyanide 3‐chlorophenylhydrazone (CCCP), a well‐known mitochondrial uncoupler, was used as a positive control for depolarization.

Because we showed that cytosolic ROS and zinc elevation begin as early as 0.5 h after MPP^+^ exposure, we next asked whether mitochondrial injury occurs even earlier. Using the mitochondria‐specific ROS probe MitoSOX, we found that mitochondrial ROS levels increased sharply—more than sixfold—within 0.5 h of MPP^+^ treatment (Figure 6C). Notably, treatment with MitoTEMPO, a mitochondria‐targeted superoxide scavenger, markedly suppressed MPP^+^‐induced neuronal death (Figure 6D).

To further determine whether mitochondrial ROS generation represents the earliest upstream event, we tested whether various inhibitors could influence MitoSOX fluorescence. As shown in Figure 6E, only MitoTEMPO significantly reduced MitoSOX signals, whereas Trolox, TPEN, leupeptin, and CA‐074 failed to attenuate mitochondrial ROS production (Figure 6E). These results indicate that neither cytosolic ROS, zinc elevation, nor lysosomal dysfunction contributes to the initial rise in mitochondrial ROS.

Taken together, these findings demonstrate that mitochondrial dysfunction—manifested by early mitochondrial ROS generation and subsequent MMP loss—constitutes the initiating event in the MPP^+^‐induced neurotoxic cascade, preceding cytosolic ROS production, zinc dysregulation, and later lysosomal damage.

### 3.7 Mitochondrial ROS and Zinc Release Mediate MPP ^+^‐Induced LMP and Cell Death

**FIGURE 7:**
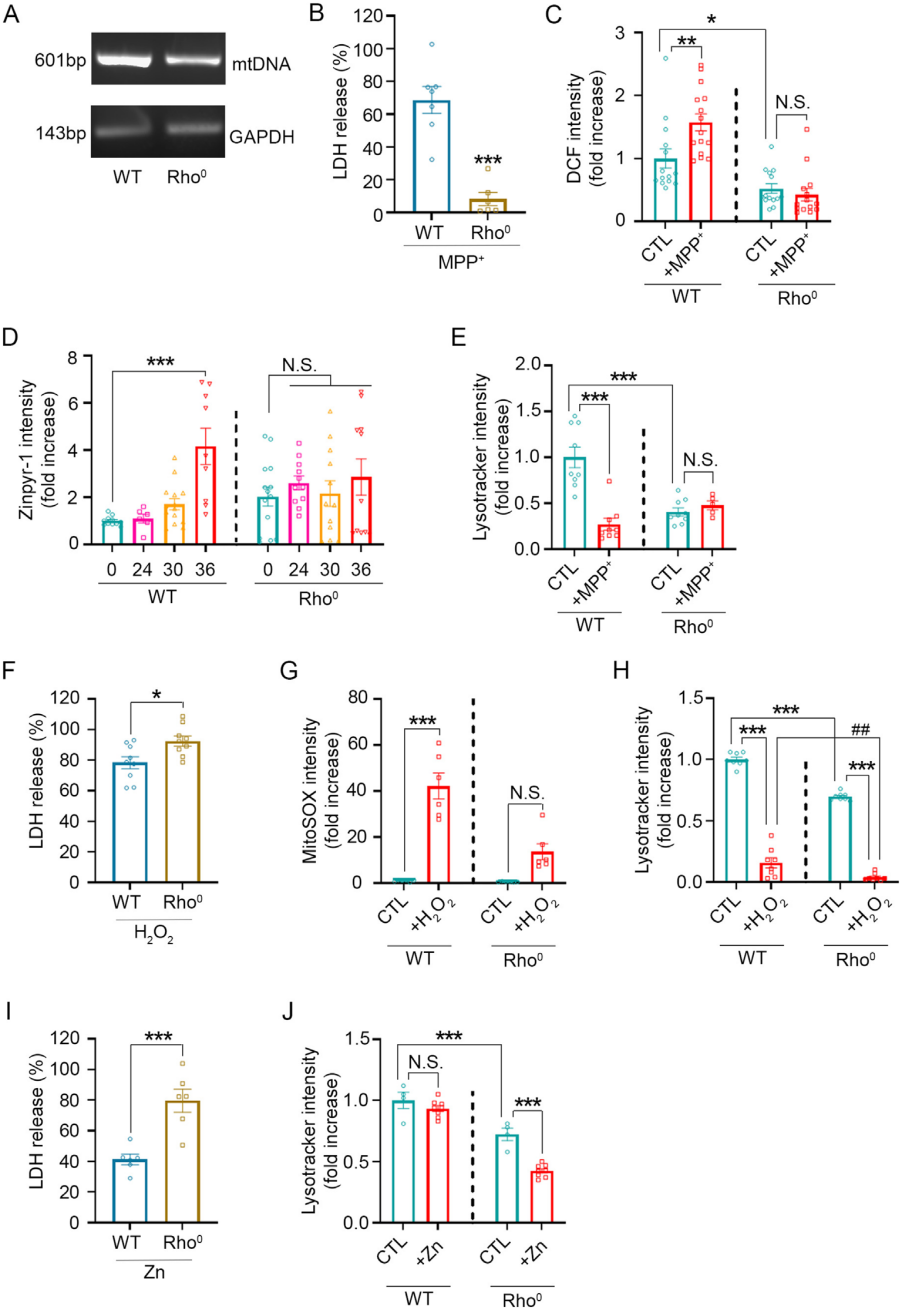
MPP^+^‐induced ROS production, zinc accumulation, LMP, and cell death are attenuated in mitochondrial DNA (mtDNA)‐depleted Rho^0^ CHO cells. (A) RT‐PCR analysis of mtDNA expression in wild‐type (WT) or Rho^0^ CHO cells, showing a marked reduction in mtDNA levels in Rho^0^ cells. (B) LDH release measured 36 h after treatment with 3 mM MPP^+^ in WT and Rho^0^ CHO cells. Data are presented as mean ± SEM. *n* = 6–7 per condition for LDH release, from 3 independent cultures with multiple technical replicates per culture. *t*(11) = 6.20, *p* < 0.0001. Statistical analysis was performed using two‐tailed Student's *t*‐test. (C) Quantification of DCF fluorescence intensity at 13 h after MPP^+^ treatment in WT and Rho^0^ CHO cells, under control or MPP^+^ treatment conditions. DCF intensity was quantified using ImageJ. Data are presented as mean ± SEM. *n* = 14–15 per condition, from 3 to 5 independent cultures with multiple technical replicates per culture. *F* (3, 53) = 19.31, *p* < 0.0001. Statistical analysis was performed using one‐way ANOVA followed by Tukey's post hoc test. (D) Quantification of Zinpyr‐1 fluorescence intensity at the indicated time points after MPP^+^ exposure in WT and Rho^0^ cells. Zinpyr‐1 intensity was quantified using ImageJ. Data are presented as mean ± SEM. *n* = 6–14 per time point for WT and 11–14 per time point for Rho^0^, from 3 independent cultures with multiple technical replicates per culture. *F* (7, 80) = 3.95, *p* = 0.0009. Statistical analysis was performed using one‐way ANOVA followed by Tukey's post hoc test. (E) Quantification of LysoTracker fluorescence at 30 h following MPP^+^ treatment in WT and Rho^0^ cells. LysoTracker fluorescence intensity was quantified using ImageJ. Data are presented as mean ± SEM. *n* = 5–9 per condition, from 3 independent cultures with multiple technical replicates per culture. *F* (3, 28) = 18.39, *p* < 0.0001. Statistical analysis was performed using one‐way ANOVA followed by Tukey's post hoc test. (F) LDH release measured 26 h after exposure to 600 μM H_2_O_2_ in WT and Rho^0^ cells. Data are presented as mean ± SEM. *n* = 9 per condition for LDH release, from 3 independent cultures with multiple technical replicates per culture. *t*(16) = 2.78, *p* = 0.0135. Statistical analysis was performed using two‐tailed Student's *t*‐test. (G) Quantification of MitoSOX fluorescence intensity at 0.5 h post‐H_2_O_2_ treatment in WT and Rho^0^ cells. MitoSOX intensity was quantified using ImageJ. Data are presented as mean ± SEM. *n* = 6 per condition, from 3 independent cultures with multiple technical replicates per culture. *F* (3, 20) = 34.61, *p* < 0.0001. Statistical analysis was performed using one‐way ANOVA followed by Tukey's post hoc test. (H) Quantification of LysoTracker fluorescence intensity at 0.5 h post‐H_2_O_2_ treatment in WT and Rho^0^ cells. LysoTracker intensity was quantified using ImageJ. Data are presented as mean ± SEM. *n* = 8 per condition, from 3 independent cultures with multiple technical replicates per culture. *F* (3, 28) = 343.0, *p* < 0.0001. Statistical analysis was performed using one‐way ANOVA followed by Tukey's post hoc test. (I) LDH release 26 h after exposure to 50 μM ZnCl_2_ in WT and Rho^0^ cells. Data are presented as mean ± SEM. *n* = 6 per condition, from 3 independent cultures with multiple technical replicates per culture. *t*(10) = 4.64, *p* = 0.0009. Statistical analysis was performed using two‐tailed Student's *t*‐test. (J) Quantification of LysoTracker fluorescence intensity at 4 h after ZnCl_2_ treatment in WT and Rho^0^ cells. LysoTracker intensity was quantified using ImageJ. Data are presented as mean ± SEM. *n* = 4–8 per condition, from 3 independent cultures with multiple technical replicates per culture. *F*(3, 20) = 66.35, *p* < 0.0001. Statistical analysis was performed using one‐way ANOVA followed by Tukey's post hoc test.

To determine whether mitochondria serve as a source of cytotoxic zinc following MPP^+^ exposure, we utilized mitochondrial DNA‐deficient Rho^0^ CHO cells. These cells lack functional oxidative phosphorylation and exhibit impaired mitochondrial membrane potential, reduced ATP production, and diminished ROS generation (Heller et al. 2013). As expected, MPP^+^‐induced cell death was markedly reduced in Rho^0^ CHO cells compared to wild‐type (WT) CHO cells (Figure 7A,B).

Consistent with these features, MPP^+^‐induced increases in ROS and intracellular zinc levels were observed only in WT cells, but not in Rho^0^ cells (Figure 7C,D). Baseline ROS levels, measured by DCF fluorescence, were approximately 50% lower in Rho^0^ cells (Figure 7C, control in WT vs. in Rho^0^), supporting the notion that mitochondria are a primary source of ROS through oxidative phosphorylation. Interestingly, despite their reduced ROS, basal cytosolic free zinc levels were nearly two‐fold higher in Rho^0^ cells (Figure 7D, 0 h in WT vs. Rho^0^ cells), suggesting that mitochondria also play a role in maintaining zinc homeostasis under physiological conditions. These findings indicate that mitochondria are involved not only in ROS production but also in maintaining intracellular zinc balance.

Supporting these observations, MPP^+^‐induced LMP was observed in WT cells but was absent in Rho^0^ cells (Figure 7E). This lack of LMP in Rho^0^ cells likely reflects the absence of MPP^+^‐induced ROS and zinc elevation. Notably, Rho^0^ cells also exhibited lower baseline lysosomal content, as indicated by reduced LysoTracker staining (Figure 7E, control in WT vs. Rho^0^), implying that mitochondrial function may be linked to lysosomal biogenesis or stability. Since both mitochondria and lysosomes contribute to cytosolic zinc buffering, dysfunction in either organelle may compromise zinc homeostasis.

Consistent with this idea, Rho^0^ cells exhibited heightened sensitivity to both oxidative stress and zinc overload (Figure 7F,I), likely due to impaired intracellular zinc‐buffering capacity. Following H_2_O_2_ treatment, WT cells showed a more than 40‐fold increase in MitoSOX fluorescence within 0.5 h, whereas Rho^0^ cells exhibited no significant mitochondrial ROS increase (Figure 7G), clearly reflecting the mitochondrial deficiency of Rho^0^ cells. Despite their markedly reduced ROS generation, Rho^0^ cells showed a more pronounced LMP response after H_2_O_2_ treatment: lysosomal content—already reduced by over 40% at baseline—declined even further (Figure 7H). This indicates that Rho^0^ cells undergo stronger LMP and higher cell death despite minimal mitochondrial ROS production.

Further supporting this conclusion, zinc overload‐induced toxicity revealed profound lysosomal vulnerability in Rho^0^ cells. Under conditions where WT cells did not yet exhibit detectable LMP, Rho^0^ cells showed a marked reduction in LysoTracker signals (Figure 7J), demonstrating that their reduced baseline lysosomal content renders them highly susceptible to zinc dyshomeostasis. Consequently, zinc overload‐induced cell death was substantially greater in Rho^0^ cells (Figure 7I).

Collectively, these findings support a model in which MPP^+^‐induced mitochondrial damage leads to ROS production and zinc release, thereby triggering lysosomal destabilization and ultimately promoting neuronal death. When mitochondrial zinc release is absent, as in Rho^0^ cells, MPP^+^‐induced LMP and cytotoxicity are significantly attenuated. Moreover, across conditions, the degree of cytotoxicity correlated strongly with the extent of LysoTracker signal loss, indicating that LMP is a key determinant of cell death.

## 4 Discussion

Our study identifies intracellular zinc as a key upstream mediator linking mitochondrial dysfunction to lysosomal membrane permeabilization (LMP) and neuronal death in MPP^+^ toxicity. While the pathological roles of zinc and reactive oxygen species (ROS) in Parkinson's disease (PD) models are well recognized, the organellar origin of zinc and its temporal relationship to lysosomal failure have remained unclear. Here, we demonstrate that mitochondria act as an early source of labile zinc during MPP^+^ exposure, and that lysosomes serve as the terminal vulnerable organelle whose destabilization leads to cell death. These findings provide a unified mechanistic framework for understanding how mitochondrial injury propagates to lysosomal dysfunction in PD‐related neurodegeneration.

Mitochondrial oxidative stress was detected as one of the earliest pathological events. MitoSOX fluorescence and lipid peroxidation increased within 30 min of MPP^+^ treatment, preceding detectable lysosomal abnormalities or cytosolic zinc elevation. In contrast, a measurable decrease in mitochondrial membrane potential (MMP), assessed by JC‐1 red/green ratios, appeared at later time points (approximately 2 h). This temporal pattern indicates that mitochondrial ROS production precedes the overt loss of MMP. Consistent with this hierarchy, only the mitochondria‐targeted antioxidant MitoTEMPO suppressed MPP^+^‐induced mitochondrial ROS, whereas Trolox, TPEN, leupeptin, and CA‐074 had no effect, supporting the conclusion that oxidative stress originates within mitochondria and is not secondarily triggered by cytosolic ROS, zinc elevation, or lysosomal damage.

The requirement of functional mitochondria for initiating this cascade was further supported by experiments using Rho^0^ CHO cells lacking mitochondrial DNA. In these cells, MPP^+^ failed to induce ROS generation, zinc elevation, LMP, or cell death to the extent observed in wild‐type cells, demonstrating that mitochondrial integrity is essential for triggering downstream zinc‐ and lysosome‐dependent toxicity. Despite this resistance to MPP^+^, Rho0 cells were markedly more sensitive to zinc overload (ZnCl_2_) and hydrogen peroxide (H_2_O_2_) and exhibited reduced LysoTracker staining, indicating diminished lysosomal abundance. Importantly, both ZnCl_2_ and H_2_O_2_ robustly induced LMP and cell death in Rho0 cells, highlighting that lysosomes are highly vulnerable once exposed to elevated zinc or oxidative stress, and that LMP is tightly correlated with cytotoxicity independent of mitochondrial function. These findings emphasize the central role of lysosomal integrity in determining cell fate.

**FIGURE 8:**
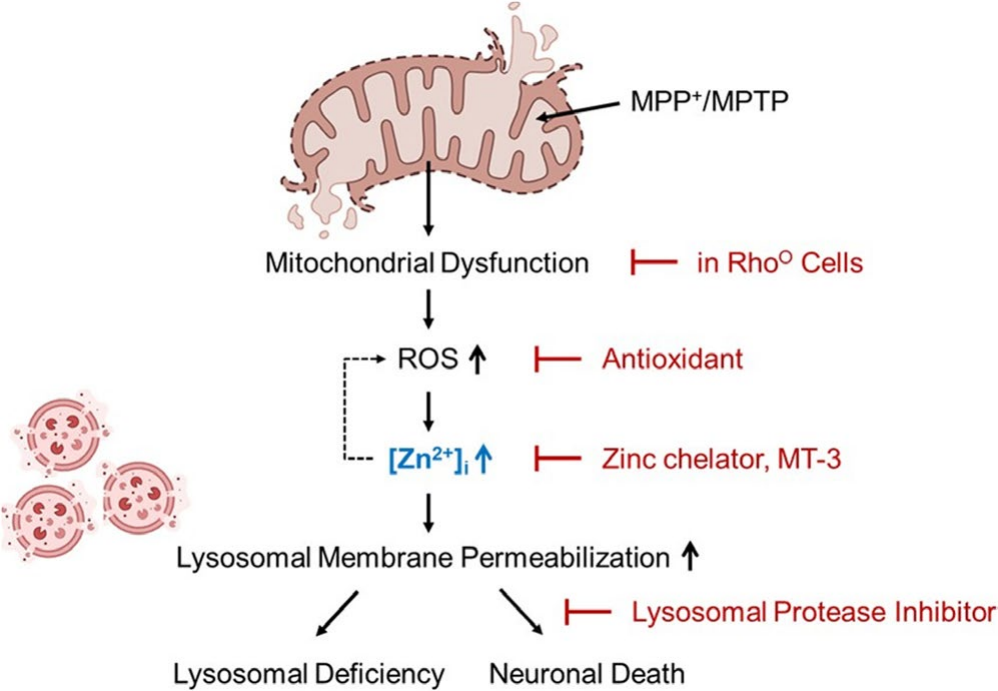
Schematic diagram illustrating the mechanism of MPP^+^‐induced neuronal death. MPP^+^ inhibits mitochondrial electron transport complex I, causing mitochondrial membrane permeabilization and increased ROS production. Mitochondrial dysfunction triggers zinc release from mitochondria, while ROS further mobilizes zinc from intracellular stores, together producing a rapid rise in cytosolic zinc levels ([Zn^2+^]ᵢ). Under physiological conditions, lysosomes—along with MT‐3—serve as the major buffering system to maintain zinc homeostasis. However, excessive ROS and the sharp elevation of cytosolic zinc exceed lysosomal zinc‐handling capacity. As lysosomes attempt to sequester the excess zinc, they become destabilized, leading to lysosomal membrane permeabilization (LMP) and loss of lysosomal integrity. The resulting leakage of lysosomal proteases into the cytoplasm ultimately drives LMP‐mediated neuronal cell death.

Our data further reveal mechanistic interactions between ROS, zinc, and LMP. Antioxidants suppressed MPP^+^‐induced zinc elevation and cathepsin B release, and zinc chelation reduced ROS levels, indicating a bidirectional amplification loop between ROS and zinc that converges on lysosomal destabilization (Figure 8). This reciprocal interplay aligns with previous reports demonstrating the cooperative nature of zinc and oxidative stress in promoting lysosomal rupture (Pratt et al. 2021; Maret 2017).

We also uncovered a context‐dependent role of metallothionein‐3 (MT‐3), a brain‐enriched zinc‐binding protein. MT‐3 deficiency increased vulnerability to MPP^+^ and zinc overload, indicating a zinc‐buffering function under endogenous mitochondrial stress. Conversely, MT‐3‐deficient astrocytes were more resistant to H_2_O_2_‐induced toxicity, consistent with the notion that MT‐3 can release zinc under exogenous oxidative conditions. These observations suggest that the directionality of zinc flux mediated by MT‐3 depends on the source and nature of oxidative stress. Considering previous reports of reduced MT expression following MPTP exposure in vivo (Dhanasekaran et al. 2008; Rojas et al. 2000), further investigation into how MT‐3 regulates zinc homeostasis under various pathological conditions will be important for understanding its role in Parkinsonian neurodegeneration.

With respect to cell death pathways, MPP^+^‐treated primary cortical neurons did not exhibit caspase‐3 activation (data not shown), and the pan‐caspase inhibitor zVAD failed to rescue cytotoxicity. These results indicate that apoptosis is not the predominant death mechanism in our conditions and further support the idea that lysosomal destabilization, rather than caspase‐dependent pathways, is the primary driver of cell death. This conclusion is consistent with previous studies showing that acute MPP^+^ exposure induces irreversible lysosomal damage distinct from mild MPP^+^ models that engage apoptotic signaling (Miyara et al. 2016).

Importantly, lysosomal dysfunction is increasingly recognized as a central feature of PD pathology. Reduced lysosomal enzyme activity and impaired autophagic flux have been reported in the substantia nigra of PD patients (Ge et al. 2021). Enhancing lysosomal biogenesis via transcription factor EB (TFEB) activation or mTOR inhibition has shown protective effects in MPTP/MPP^+^ models (Liu et al. 2013; Torra et al. 2018). In this study, we explore the role of zinc as a critical mediator between mitochondrial damage and lysosomal depletion. While previous research has reported direct permeabilization of lysosomal membranes, leading to LMP, by the pro‐apoptotic protein BAX (BCL2‐associated X protein) in MPP^+^‐treated cells (Bové et al. 2014), our findings demonstrate that zinc released from mitochondria induces LMP and resultant lysosomal depletion. Thus, further investigation is required to examine whether translocation and internalization of BAX into lysosomes are related to zinc.

In summary, our results establish a mechanistic cascade in which MPP^+^ induces early mitochondrial ROS generation, followed by mitochondrial zinc release, subsequent LMP, and ultimately neuronal death. We show that mitochondria function not only as a major source of ROS but also as an essential reservoir of zinc, and that lysosomes act as downstream organelles whose susceptibility to zinc overload determines cellular outcome. The dual impairment of mitochondrial and lysosomal zinc‐buffering capacity—observed both in MPP^+^‐treated neurons and in mtDNA‐deficient cells—highlights the importance of organellar coordination in maintaining zinc homeostasis. These findings reveal a mitochondria‐zinc–lysosome axis that may contribute to neurodegeneration and identify zinc regulation and lysosomal stabilization as promising therapeutic targets for Parkinson's disease. Further studies in in vivo models will be essential to determine the extent to which this pathway contributes to progressive dopaminergic neuron loss and to identify key molecular regulators of mitochondrial zinc release and lysosomal vulnerability.

## 5 Conclusions

This study delineates a zinc‐mediated pathological cascade that links mitochondrial dysfunction to lysosomal destabilization in MPP^+^‐induced neurotoxicity. Early mitochondrial oxidative stress leads to the release of labile zinc into the cytosol, which in turn promotes lysosomal membrane permeabilization and cathepsin leakage, ultimately driving neuronal death. These events are accompanied by a reduction in lysosomal content, indicating that mitochondrial injury disrupts lysosomal homeostasis as a downstream consequence.

Experiments using mtDNA‐deficient Rho^0^ cells further underscore the central role of mitochondria in initiating this cascade. In the absence of functional mitochondria, MPP^+^ failed to induce ROS elevation, zinc mobilization, lysosomal damage, or cytotoxicity. However, Rho^0^ cells showed heightened vulnerability to exogenous zinc and ROS, together with reduced lysosomal abundance, revealing that both mitochondria and lysosomes are integral to maintaining intracellular zinc buffering capacity. When either system is compromised, the susceptibility to zinc‐driven lysosomal failure markedly increases.

Collectively, these findings establish cytosolic zinc as a key integrator of mitochondrial and lysosomal dysfunction and support a model in which impaired mitochondrial integrity renders lysosomes highly vulnerable to zinc overload, thereby exacerbating neurodegenerative processes. Targeting zinc signaling or reinforcing lysosomal resilience may therefore represent promising therapeutic approaches for mitigating mitochondrial–lysosomal dysfunction in PD.

## Author Contributions

**Hyun‐Seung Lee:** investigation, methodology, formal analysis, writing – original draft. **Sun‐Ah Kang:** investigation, methodology, formal analysis. **Jae‐Won Eom:** methodology, investigation, formal analysis. **Min Seong Kim:** methodology, investigation, formal analysis, writing – original draft. **Ji‐Soo Kim:** methodology, formal analysis, investigation, writing – original draft. **Yang‐Hee Kim:** supervision, writing – original draft, writing – review and editing, conceptualization, formal analysis.

## Funding

This work was supported by National Research Foundation of Korea (NRF) grants RS‐2023‐00242206 and RS‐2025‐00560220; Korea Basic Science Institute (National Research Facilities and Equipment Center) grants RS‐2023‐NF001356; and the Seoul RISE Center grant 2025‐RISE‐01‐019‐04.

## Ethics Statement

The animal study protocol was approved by the Animal Care and Use Committee of Sejong University (SJ‐20230119) and was conducted following the guidelines of the Care and Use of Laboratory Animals.

## Conflicts of Interest

The authors declare no conflicts of interest.

## Supporting information


**Figures S1–S2:** jnc70363‐sup‐0001‐FiguresS1‐S2.docx.

**Appendix S1:** jnc70363‐sup‐0002‐AppendixS1.xlsx.

## Data Availability

The data that support the findings of this study are available on request from the corresponding author. The data are not publicly available due to privacy or ethical restrictions.
