# Supplementary material for: Zinc‐Mediated Lysosomal Destabilization Links Mitochondrial Damage to Neuronal Death in a Cellular MPP + Model of Parkinson's Disease
Source: J Neurochem. 2026 Feb 1;170(2):e70363. doi: 10.1111/jnc.70363 (PMC12862199; doi:10.1111/jnc.70363)
Supplement: Supplementary file 1 — Figures S1–S2: jnc70363‐sup‐0001‐FiguresS1‐S2.docx. [file JNC-170-0-s002.docx]

**Zinc-mediated lysosomal destabilization links mitochondrial damage to neuronal death in a cellular MPP^+^ model of Parkinson’s disease**

Hyun-Seung Lee^1,2^, Sun-Ah Kang^1^, Jae-Won Eom^1^, Min Seong Kim^1, 3^, Ji-Soo Kim^1^ and Yang-Hee Kim^1,3*^

^1^Department of Integrative Bioscience and Biotechnology, Sejong University, Seoul 05006, Republic of Korea

^2^Zincure Corp., Seoul 05006, Republic of Korea

^3^Institute of Bioscience and Biotechnology, Sejong University, Seoul 05006, Republic of Korea

**^*^Correspondence to:** Yang-Hee Kim, PhD

E-mail: [yhkim@sejong.ac.kr](mailto:yhkim@sejong.ac.kr)

**
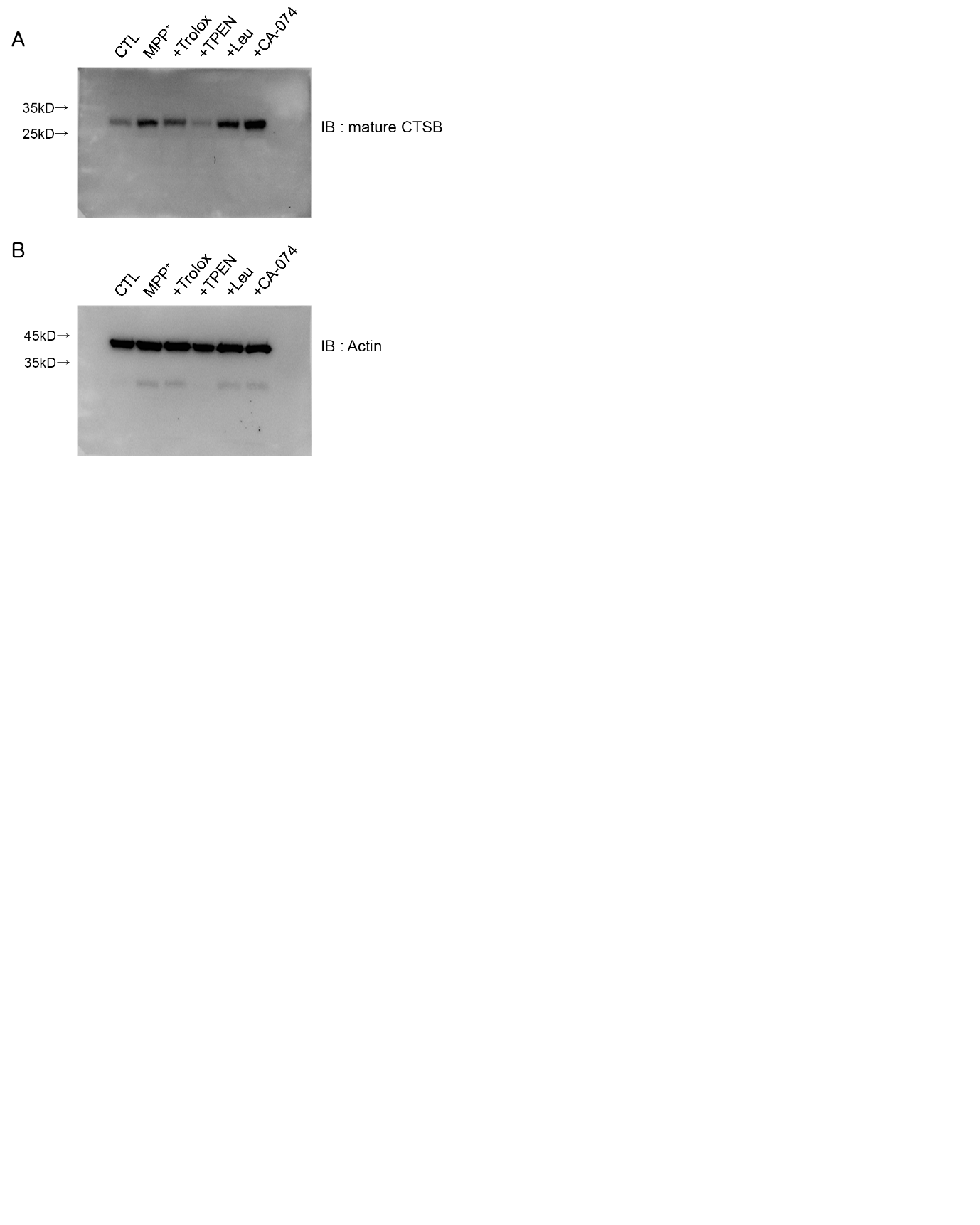
FIGURE S1.** Image of full immunoblots of cytosolic mature Cathepsin B (A) and Actin (B) corresponding to Figure 3D


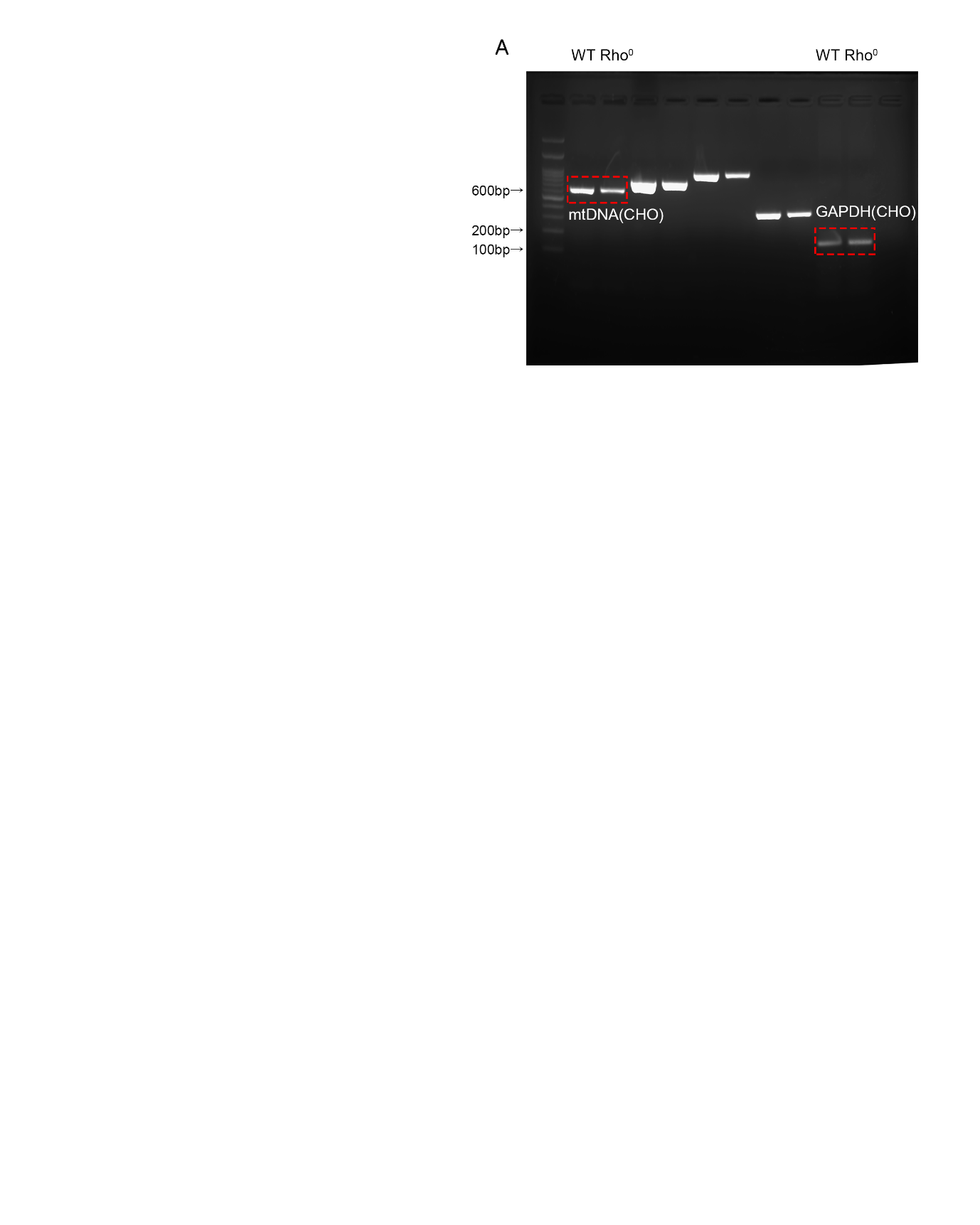


**FIGURE S2.** Image of PCR products of mtDNA and GAPDH (A) corresponding to Figure 7A
